# Hyperglycemia Aggravates 6‐Hydroxydopamine‐Induced Neuronal Ferroptosis via SLC7A11‐Dependent Pathway in Diabetic PD Rat Model

**DOI:** 10.1111/cns.70487

**Published:** 2025-07-04

**Authors:** Ya Zhao, Dan Wang, Yanwei Wang, Dan Mu, Lang Qu, Rong Li

**Affiliations:** ^1^ Medical School of Ophthalmology and Optometry North Sichuan Medical College Nanchong Sichuan China; ^2^ Institute of Basic Medicine North Sichuan Medical College Nanchong Sichuan China; ^3^ Department of Pharmacology, College of Medicine Jiaxing University Jiaxing China; ^4^ Library & Archives of North Sichuan Medical College North Sichuan Medical College Nanchong China; ^5^ School of Pharmacy North Sichuan Medical College Nanchong Sichuan China; ^6^ Department of Ophthalmology Affiliated Hospital of North Sichuan Medical College Nanchong Sichuan China

**Keywords:** diabetes‐Parkinson's disease association, ferroptosis, glutathione metabolism, hyperglycemia‐induced neurodegeneration, SLC7A11

## Abstract

**Background:**

The epidemiological link between diabetes mellitus (DM) and Parkinson's disease (PD) is well‐established, but the mechanistic basis remains unclear. Chronic hyperglycemia, a hallmark of DM, may exacerbate PD pathogenesis, though the underlying molecular pathways are poorly defined.

**Methods:**

Using an integrative approach combining metabolomic profiling, proteomic analysis, and molecular characterization in vitro and in vivo models, we investigated the role of the cystine/glutamate antiporter system in glucose‐induced neuronal vulnerability. SLC7A11 expression was genetically restored, and adeno‐associated viral vectors delivered SLC7A11 to the nigrostriatal pathway in a streptozotocin‐induced diabetic PD rat model to evaluate neuroprotection.

**Results:**

Chronic high glucose impaired SLC7A11 function, reducing cystine uptake and depleting intracellular glutathione in dopaminergic neurons, increasing susceptibility to 6‐hydroxydopamine‐induced ferroptosis. SLC7A11 restoration rescued neuronal viability, restored redox homeostasis, and attenuated motor deficits and dopaminergic neuron loss in the diabetic PD model. Mechanistically, SLC7A11 enhanced glutathione synthesis and suppressed ferroptosis signaling pathways.

**Conclusion:**

Chronic hyperglycemia disrupts the cystine/SLC7A11/glutathione axis, accelerating neuronal degeneration and linking DM to PD susceptibility. SLC7A11 emerges as a potential therapeutic target to mitigate neurodegeneration in diabetic individuals at risk for PD.

## Graphical abstract

Chronic hyperglycemia disrupts the cystine/SLC7A11/glutathione axis, impairing cystine uptake and depleting glutathione in dopaminergic neurons, increasing ferroptosis susceptibility. SLC7A11 restoration rescues neuronal viability, restores redox balance, and attenuates neurodegeneration in diabetic PD models. SLC7A11 emerges as a therapeutic target to mitigate PD risk in diabetic individuals.

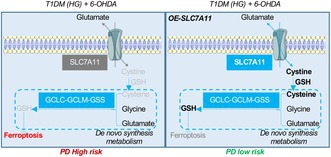

**Abbreviations**
4‐HNE: 4‐hydroxynonenal.
AP: amphetamine
DHS: donor horse serum
DM: diabetes mellitus
Factor TEM: transmission electron microscopy
FAS: forepaw adjusting steps
GSH Streptozotocin: glutathione STZ
HG: high glucose
MFB: medial forebrain bundle
NGF: nerve growth
PD: Parkinson's disease
SN: substantia nigra

## 1 Introduction

The intricate relationship between metabolic disorders and neurodegenerative diseases has garnered increasing attention in recent years. Diabetes mellitus (DM), characterized by chronic hyperglycemia, has emerged as a significant risk factor for Parkinson's disease (PD), with compelling epidemiological evidence demonstrating a bidirectional association between these conditions [1, 2, 3, 4, 5, 6]. This interconnection is further supported by shared pathophysiological features, including protein misfolding, lysosomal dysfunction, mitochondrial impairment, and chronic low‐grade inflammation [7]. Notably, the coexistence of DM and PD appears to exacerbate disease progression, with clinical studies reporting more severe postural instability, gait disturbances, and faster L‐DOPA dosage escalation in PD patients with comorbid DM [8, 9, 10, 11, 12, 13]. Preclinical models have provided mechanistic insights, revealing that DM exacerbates striatal oxidative stress, disrupts dopaminergic neurotransmission, and enhances susceptibility to neurodegeneration in PD [14]. However, the precise molecular mechanisms underlying this pathological synergy remain incompletely understood.

Central to this DM‐PD interplay is the role of chronic hyperglycemia, which exerts profound effects on neuronal homeostasis. The brain's high metabolic demand and limited glucose storage capacity render it particularly vulnerable to glycemic fluctuations. Hyperglycemia‐induced pathologies, including oxidative stress [15, 16, 17, 18], advanced glycation end product formation [15, 19, 20], neuroinflammation [21, 22], and neurotoxicity [23, 24], collectively contribute to dopaminergic neuron vulnerability. The neuroprotective effects of hypoglycemic agents, particularly DPP4 inhibitors and GLP‐1 analogues, further underscore the critical role of glycemic control in PD risk modulation [25]. Our previous work demonstrated that high glucose (HG) exposure exacerbates neuronal susceptibility to 6‐hydroxydopamine (6‐OHDA) toxicity in diabetic rodent models [26], yet the precise molecular mechanisms underlying this phenomenon remain to be fully elucidated.

In the present study, we identify a novel mechanism through which HG exacerbates PD pathogenesis. We demonstrate that HG impairs SLC7A11‐mediated cystine transport, leading to glutathione (GSH) depletion and enhanced susceptibility to 6‐OHDA‐induced ferroptosis. This discovery not only provides mechanistic insights into the DM‐PD connection but also identifies potential therapeutic targets for mitigating neurodegeneration in diabetic individuals at risk for PD. Our findings build upon previous research to offer a comprehensive understanding of how metabolic dysregulation contributes to neurodegenerative processes, potentially informing new strategies for PD prevention and management in the context of diabetes.

## 2 Materials and Methods

### 2.1 Animal Models and Ethical Considerations

Male Sprague–Dawley rats (15–20 weeks old, 300–350 g) were housed in individually ventilated cages under specific pathogen‐free conditions at 22°C ± 1°C with 50%–60% relative humidity and a 12‐h light/dark cycle. Standard rodent chow and autoclaved water were provided ad libitum. All procedures were approved by the Institutional Animal Care and Use Committee of North Sichuan Medical College (approval number NSMC 2024108) and conducted in accordance with ARRIVE guidelines 2.0 and the NIH Guide for the Care and Use of Laboratory Animals.

### 2.2 Induction of Diabetes Mellitus and Insulin Administration

Diabetes was induced by intraperitoneal injection of streptozotocin (STZ; 50 mg/kg body weight) dissolved in 0.1 M citrate buffer (pH 4.5) for five consecutive days. Control animals received citrate buffer. Fasting blood glucose levels were monitored 72 h post‐injection using a glucometer (Accu‐Chek Performa Nano; Roche). Rats with sustained non‐fasting blood glucose levels > 250 mg/dL on three consecutive measurements were considered diabetic. Body weight and blood glucose levels were monitored weekly.

After a 3‐day recovery period, rats received daily intraperitoneal injections of insulin (4 U/kg body weight) or vehicle (0.9% NaCl) for 4 weeks to evaluate chronic effects [26].

### 2.3 6‐OHDA Lesion and Viral Vector Delivery

Rats were anesthetized with 2% isoflurane in oxygen (1 L/min) and positioned in a stereotaxic frame. 6‐OHDA (2 μg/μL in 0.9% NaCl with 0.2% ascorbic acid) was injected unilaterally into the medial forebrain bundle (MFB; coordinates: AP = −3.2 mm, L = −1.5 mm, DV = −8.7 mm) at 0.5 μL/min using a Hamilton syringe. The needle was left in place for 10 min before withdrawal.

For viral delivery, AAV9 vectors expressing mCherry‐control (pAVV‐CMV‐mcherry‐3xFLAG‐WPRE) or SLC7A11‐mCherry (pAVV‐CMV‐mcherry‐P2A‐SLC7A11‐3xFLAG‐WPRE) were injected into the MFB using the same coordinates. Viral expression was confirmed by fluorescence microscopy and Western blot analysis after 4 weeks.

### 2.4 Behavioral Assessments

Behavioral assessments, including the rotarod test, forepaw adjusting steps (FAS) test, cylinder test, and amphetamine‐induced rotation, were performed 4 weeks post‐6‐OHDA injection. All tests were conducted between 9:00 AM and 5:00 PM by investigators blinded to treatment groups. Detailed methodologies are provided in the Text S1.

### 2.5 Immunohistochemistry

Prior to histological processing, all experimental animals underwent comprehensive motor function assessments. For immunohistochemical characterization, 5‐μm coronal brain sections were prepared from paraffin‐embedded tissue samples. These sections were subjected to overnight incubation at 4°C with a rabbit polyclonal anti‐tyrosine hydroxylase primary antibody (TH; 1:500 dilution; Proteintech, Cat# 25859‐1‐AP). Immunodetection was achieved through a standard immunoperoxidase protocol using 3,3′‐diaminobenzidine (DAB) as the chromogenic substrate. The immunostained sections were examined using a Nikon ECLIPSE50i brightfield microscope, with high‐resolution digital images systematically acquired for subsequent quantitative and qualitative analysis.

### 2.6 Cell Culture, Differentiation, and Viability

PC12 cells were cultured in RPMI‐1640 medium supplemented with 10% heat‐inactivated horse serum, 5% fetal bovine serum, and 1% penicillin–streptomycin at 37°C in a 5% CO_2_ atmosphere. Cells were passaged every 3–4 days at ~80% confluence using 0.25% trypsin–EDTA and used between passages 4 and 15.

For differentiation, cells were incubated in RPMI‐1640 medium containing2 mM L‐glutamine, 50 μg/mL gentamicin, 2.5 μg/mL amphotericin B, 1% donor horse serum, and 100 ng/mL nerve growth factor (NGF; Sigma‐Aldrich). Medium was refreshed every 48 h, and differentiation was confirmed by phase‐contrast microscopy based on neurite outgrowth and cellular elongation. Cell viability was assessed using the sulforhodamine B (SRB; Sigma‐Aldrich) assay. After treatment, cells were fixed with 10% (w/v) cold trichloroacetic acid at 4°C for 1 h, stained with 0.4% (w/v) SRB in 1% acetic acid for 30 min at room temperature, and washed with 1% acetic acid. Protein‐bound dye was solubilized with 10 mM Tris base (pH 10.5), and absorbance was measured at 565 nm using a microplate reader (SpectraMax M5, Molecular Devices). Viability was calculated as: (A565 treated/A565 DMSO control) × 100%. Experiments were performed in triplicate and repeated three times independently. Data are expressed as mean ± SD of three biological replicates.

### 2.7 Plasmid Transfection

For SLC7A11 overexpression, PC12 cells at 60%–70% confluence were transfected with either a control vector (pSLenti ‐EF1‐mCherry‐P2A‐Puro‐CMV‐MCS‐3Xflag‐WPRE) or an SLC7A11 overexpression construct (pSLenti‐EF1‐mCherry‐P2A‐Puro‐CMV‐SLC7A11‐3Xflag‐WPRE) using UltraFection 3.0 transfection reagent (Elji Biotechnology). The DNA‐lipid complex (1:2 DNA‐to‐reagent ratio) was incubated with cells in serum‐free medium for 6 h at 37°C, followed by replacement with complete growth medium. Transfection efficiency was assessed 24 h post‐transfection via mCherry fluorescence using fluorescence microscopy.

### 2.8 L‐ROS Detection

After treatment, cells were washed twice with PBS (137 mM NaCl, 2.7 mM KCl, 10 mM Na_2_HPO_4_, 1.8 mM KH_2_PO_4_, pH 7.4) and incubated with 2 μM C11‐BODIPY 581/591 (Thermo Fisher Scientific) in serum‐free medium at 37°C in a 5% CO_2_ atmosphere for 20 min. Cells were then washed twice with PBS, fixed with 4% paraformaldehyde for 15 min at room temperature, and mounted on glass slides using antifade mounting medium with DAPI (Thermo Fisher Scientific) for nuclear staining.

Fluorescence images were captured using a Nikon ECLIPSE 50i microscope equipped with a DS‐Qi2 CCD camera and NIS‐Elements software (version 5.21). Fluorescence intensity was quantified using ImageJ (version 1.53t, NIH), with at least five random fields analyzed per sample.

### 2.9 Metabolomics Analysis

The detailed procedures for metabolite extraction, analysis, and identification are outlined in Text S1 within the Supporting Information S1.

### 2.10 Proteomics Analysis

The detailed procedures for protein extraction, examination, and recognition are outlined in Text S1.

### 2.11 Biochemical Analysis

#### 2.11.1 GSH and GSSG

Cellular lysates and substantia nigra tissues were homogenized in ice‐cold RIPA buffer (50 mM Tris–HCl, pH 7.4, 150 mM NaCl, 1% NP‐40, 0.5% sodium deoxycholate, 0.1% SDS) containing a protease inhibitor cocktail (Thermo Fisher Scientific). Protein concentrations were determined using the Pierce BCA Protein Assay Kit (Thermo Fisher Scientific) with bovine serum albumin as the standard.

For glutathione measurement, reduced (GSH) and oxidized (GSSG) glutathione levels were quantified using a GSH/GSSG Assay Kit (Beyotime Biotechnology). Samples were deproteinized with 5% metaphosphoric acid and centrifuged at 10,000×*g* for 10 min at 4°C. Supernatants were analyzed following the manufacturer's protocol, with absorbance measured at 412 nm using a microplate reader (SpectraMax M5, Molecular Devices). GSH and GSSG levels were normalized to total protein content (nmol/mg protein), and the GSH/GSSG ratio was calculated to assess oxidative stress. All experiments were performed in triplicate and repeated three times independently.

#### 2.11.2 MDA

Substantia nigra tissues were homogenized in ice‐cold RIPA buffer (50 mM Tris–HCl, pH 7.4, 150 mM NaCl, 1% NP‐40, 0.5% sodium deoxycholate, and 0.1% SDS) supplemented with a protease inhibitor cocktail (Thermo Fisher Scientific). Protein concentrations were determined using the Pierce BCA Protein Assay Kit (Thermo Fisher Scientific) with bovine serum albumin as the standard.

MDA levels, reflecting lipid peroxidation, were measured using a Lipid Peroxidation MDA Assay Kit (Beyotime Biotechnology). Tissue homogenates were mixed with thiobarbituric acid (TBA) reagent, heated at 95°C for 60 min, and centrifuged at 10,000×*g* for 10 min at 4°C. Absorbance of the supernatant was measured at 532 nm using a microplate reader (SpectraMax M5, Molecular Devices). MDA concentrations were calculated using a 1,1,3,3‐tetramethoxypropane standard curve and normalized to total protein content (nmol MDA/mg protein). All experiments were performed in triplicate and repeated three times independently.

### 2.12 Transmission Electron Microscopy

TEM analysis was conducted at the Cryo‐Electron Microscopy Center of Zhejiang University. Cells were fixed in 2.5% glutaraldehyde in 0.1 M phosphate buffer (pH 7.3) for 72 h at 4°C, followed by three washes with 0.1 M PBS. Post‐fixation was performed with 1% osmium tetroxide in 0.1 M PBS for 1 h at room temperature, and samples were stained en bloc with 2% uranyl acetate in 50% ethanol for 30 min at 4°C.

Dehydration was carried out using a graded ethanol series (30%, 50%, 70%, 90%, and 100%) for 15 min each, followed by two changes of pure acetone. Samples were infiltrated with a 1:1 (v/v) mixture of acetone and Spurr's low‐viscosity embedding medium (Electron Microscopy Sciences) for 2 h at 25°C, then embedded in pure medium overnight. Polymerization was performed at 60°C for 48 h.

Ultra‐thin sections (70 nm) were cut using a Leica UC7 ultramicrotome (Leica Microsystems) and collected on 200‐mesh copper grids. Sections were counterstained with 2% uranyl acetate for 10 min and lead citrate for 5 min. Images were acquired using a Tecnai T10 transmission electron microscope (Thermo Fisher Scientific) operated at 80 kV, equipped with a Gatan Orius SC1000 CCD camera (Gatan).

### 2.13 Western Blotting

Cell lysates or brain tissue homogenates were prepared using RIPA lysis buffer (Cell Signaling Technology) supplemented with protease and phosphatase inhibitor cocktails (Thermo Fisher Scientific). Protein concentrations were determined using the Pierce BCA Protein Assay Kit (Thermo Fisher Scientific) with bovine serum albumin as the standard.

Equal amounts of protein (40 μg per lane) were separated by 10% SDS‐PAGE and transferred onto PVDF membranes (Millipore). Membranes were blocked with 5% non‐fat dry milk in TBST for 1 h at room temperature and incubated overnight at 4°C with primary antibodies: anti‐GCLC (1:2000, Proteintech), anti‐GCLM (1:2000), anti‐GSS (1:2000), anti‐SLC7A11 (1:2000), and anti‐β‐actin (1:5000). After washing, membranes were incubated with HRP‐conjugated secondary antibodies (1:5000, Cell Signaling Technology) for 1 h at room temperature.

Protein bands were visualized using Ultra ECL Western Blotting Detection Reagent (Yeasen Biotechnology) and imaged with a ChemiDoc MP Imaging System (Bio‐Rad). Band intensities were quantified using ImageJ (NIH), and target protein expression was normalized to β‐actin. Experiments were performed in triplicate and repeated three times independently.

### 2.14 Bioinformatics Analysis of SLC7A11 Expression in DM and PD

To explore the association between SLC7A11 expression and DM/PD, we analyzed gene expression datasets from the GEO database. For PD, datasets GSE20314 (4 PD cases, 4 controls) and GSE88888 (10 PD cases, 10 controls) were used. For DM, datasets GSE55098 and GSE101931 were combined, yielding 30 DM cases and 30 controls.

Raw data were processed in R. Probe IDs were converted to gene symbols using platform‐specific annotations, and data were normalized using the robust multi‐array average (RMA) algorithm. Batch effects were corrected using the ComBat function. Differential expression analysis of SLC7A11 was performed using the limma package, with significance thresholds set at adjusted *p* < 0.05 and |log2 fold change| > 0.5.

Data visualization was conducted using ggplot2 and pheatmap. The analysis revealed significant differences in SLC7A11 expression between disease and control groups across all datasets. Statistical analyses were performed in RStudio.

### 2.15 Tatistical Analyses

All statistical analyses were performed using R software. Data distribution normality was verified using the Shapiro–Wilk test, while homogeneity of variance was assessed with Levene's test. For comparisons among multiple groups, we employed one‐way analysis of variance (ANOVA) followed by: Dunnett's post hoc test when comparing multiple treatments against a single control group and Bonferroni‐adjusted pairwise comparisons for all possible group comparisons. Results are presented as mean ± standard error of the mean (SEM) from a minimum of three independent biological replicates. Statistical significance thresholds were established as follows: *p < 0.05, **p < 0.01, and ***p < 0.001. All hypothesis tests were conducted as two‐tailed analyses, with appropriate corrections for multiple comparisons where applicable. Data visualization was performed using the ggplot2 package within the R environment.

## 3 Results

### 3.1 High Glucose Potentiates 6‐OHDA‐Induced Neuronal Ferroptosis Through Enhanced Lipid Peroxidation and Mitochondrial Dysfunction

6‐OHDA, a dopamine analog with selective neurotoxic properties, is widely utilized to establish both in vivo and in vitro models of advanced PD. Mechanistic studies have demonstrated that 6‐OHDA induces ferroptosis in dopaminergic neurons, leading to degeneration of the nigrostriatal pathway and subsequent motor dysfunction [27]. Previous investigations have revealed that subthreshold doses of 6‐OHDA can specifically damage the dopaminergic pathway in the nigrostriatal region of diabetic rodents [26], suggesting that diabetes may exacerbate the susceptibility of nigrostriatal dopaminergic neurons to 6‐OHDA‐induced ferroptosis.

**FIGURE 1:**
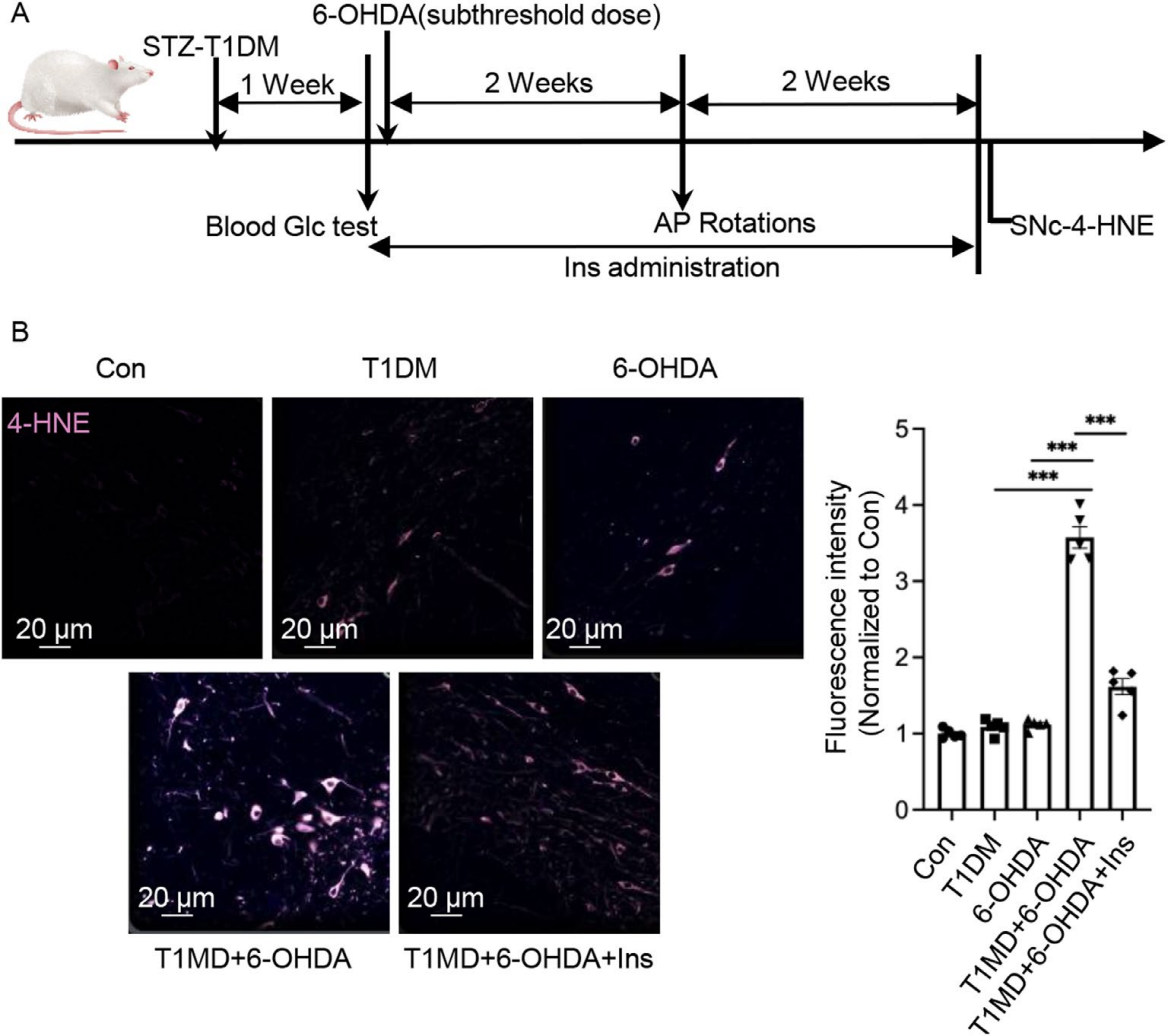
Insulin attenuates 6‐OHDA‐induced lipid peroxidation in the substantia nigra of diabetic rats. (A) Schematic representation of the experimental timeline and treatment protocol. (B) Representative immunofluorescence images (left) of coronal sections from the substantia nigra pars compacta (SNc) in non‐diabetic, streptozotocin (STZ)‐induced diabetic, and insulin‐treated diabetic rats, stained for 4‐hydroxynonenal (4‐HNE), a biomarker of lipid peroxidation. Scale bar: 20 μm. Quantitative analysis of 4‐HNE fluorescence intensity (right) in the SNc. Data are presented as mean ± SEM. *n* = 5 biologically independent samples. Statistical analysis was performed using one‐way ANOVA followed by Tukey's multiple comparisons test. ***p < 0.001. 4‐HNE, 4‐hydroxynonenal; 6‐OHDA, 6‐hydroxydopamine; AP, amphetamines; Glc, glucose; Ins, insulin; SD‐Rat, Sprague–Dawley rats; SN, substantia nigra; STZ, streptozotocin; T1DM, type 1 diabetes mellitus.

To test this hypothesis, we utilized the experimental paradigm outlined in Figure 1A. To assess 6‐OHDA‐induced ferroptosis in nigral dopaminergic neurons of diabetic rats, we quantified 4‐hydroxynonenal (4‐HNE), a stable end product of lipid peroxidation and a widely recognized biomarker of oxidative stress. Immunohistochemical analysis demonstrated a marked increase in 4‐HNE immunoreactivity within dopaminergic neurons of the substantia nigra (SN) in diabetic rats exposed to 6‐OHDA, compared to those subjected to either diabetes or 6‐OHDA treatment alone (Figure 1B,C). Notably, insulin administration significantly attenuated the number of 4‐HNE‐positive dopaminergic neurons in the SN of T1DM + 6‐OHDA rats relative to untreated T1DM + 6‐OHDA controls. Collectively, these findings strongly implicate a hyperglycemic microenvironment in the central nervous system as a critical risk factor that enhances the susceptibility of substantia nigra dopaminergic neurons to 6‐OHDA‐induced ferroptosis.

**FIGURE 2:**
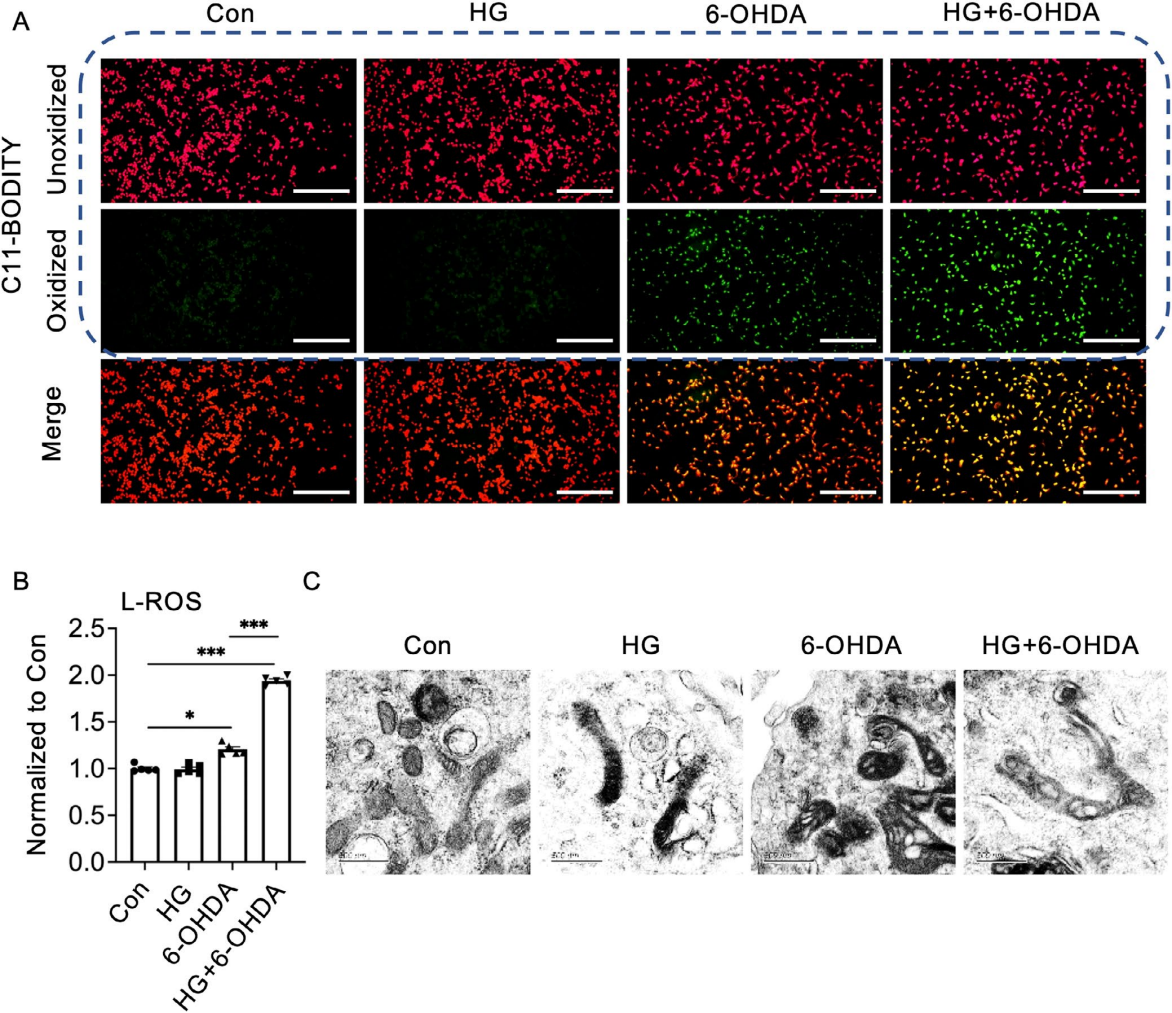
High glucose (HG) exacerbates 6‐OHDA‐induced ferroptosis in neuronal cells in vitro. NGF‐differentiated PC12 cells were cultured under HG (30 mM glucose) conditions with or without 50 μM 6‐OHDA. (A) Lipid peroxidation was assessed using C11‐BODIPY 581/591. Fluorescent microscopy images show oxidized BODIPY C11 (green) and unoxidized BODIPY C11 (red). Scale bars: 200 μm. (B) Levels of lipid reactive oxygen species (L‐ROS) were quantified using a microplate reader. (C) Representative transmission electron microscopy (TEM) images illustrating mitochondrial morphology. Scale bars: 500 nm. Data are presented as mean ± SEM; *n* = 5 biologically independent samples. Statistical analysis was performed using one‐way ANOVA followed by Tukey's multiple comparisons test. *p < 0.05, ***p < 0.001. 6‐OHDA, 6‐hydroxydopamine; Glc, glucose; L‐ROS, lipid reactive oxygen species; NGF, nerve growth factor; NGF‐PC12 cells, PC12 cells differentiated by nerve growth factor.

To investigate whether HG potentiates 6‐OHDA‐induced neuronal ferroptosis, we employed the C11‐BODIPY 581/591 fluorescent probe, a sensitive indicator of intracellular lipid peroxidation that specifically detects the formation of lipid peroxides during ferroptosis. Quantitative analysis revealed that 6‐OHDA treatment alone induced a moderate but significant increase in intracellular lipid peroxide levels. While HG treatment alone did not significantly alter basal lipid peroxide levels, it markedly enhanced 6‐OHDA‐induced lipid peroxidation, as demonstrated by increased fluorescence intensity of oxidized C11‐BODIPY (Figure 2A,B). These findings suggest that HG exacerbates 6‐OHDA‐induced ferroptosis by amplifying lipid peroxidation, a hallmark feature of this iron‐dependent form of regulated cell death.

In addition to intracellular lipid peroxidation, ferroptosis is characterized by distinct mitochondrial morphological alterations, including mitochondrial shrinkage, increased membrane density, and the reduction or complete loss of mitochondrial cristae. To investigate the effects of HG on 6‐OHDA‐induced mitochondrial structural changes, we conducted transmission electron microscopy (TEM) analysis. The results revealed that neurons treated with 6‐OHDA alone exhibited characteristic ferroptotic mitochondrial changes, including a reduction in mitochondrial volume, increased double membrane thickness, and partial loss of cristae structure. While HG treatment alone resulted in elevated mitochondrial membrane density without significantly altering overall morphology, the combination of HG and 6‐OHDA treatment exacerbated mitochondrial damage, leading to near‐complete cristae disintegration and severe disruption of double membrane integrity (Figure 2C). These ultrastructural changes provide compelling evidence that HG potentiates 6‐OHDA‐induced ferroptosis through mitochondrial dysfunction.

To establish a causal relationship between ferroptosis and neuronal damage induced by HG and 6‐OHDA co‐treatment, we employed a pharmacological intervention approach using ferrostatin‐1 (Fer‐1), a specific ferroptosis inhibitor that prevents lipid peroxide accumulation through radical‐trapping antioxidant activity. Quantitative analysis demonstrated that Fer‐1 treatment (1 μM) significantly reduced intracellular lipid peroxide levels in HG + 6‐OHDA‐treated neurons, as measured by C11‐BODIPY fluorescence intensity (Figure S1A). Consistent with these findings, Fer‐1 pretreatment markedly attenuated HG + 6‐OHDA‐induced cellular damage, improving cell viability as assessed by the SRB colorimetric assay (Figure S1B). These results provide mechanistic evidence that ferroptosis plays a central role in mediating neuronal damage under conditions of combined HG and 6‐OHDA exposure. These results establish a critical role for HG in promoting ferroptotic cell death in dopaminergic neurons exposed to 6‐OHDA, suggesting that hyperglycemic conditions may create a permissive environment for ferroptosis in the context of PD pathology.

### 3.2 High Glucose Exacerbates 6‐OHDA‐Induced Dysregulation of Glutathione Homeostasis and Antioxidant Defense Systems

Ferroptosis, an iron‐dependent form of regulated cell death, is characterized by the accumulation of lipid peroxides and is intricately linked to cellular metabolic processes [28]. The core mechanism involves the peroxidation of polyunsaturated fatty acid‐containing phosphatidylethanolamines, leading to membrane destabilization and subsequent cell death. This process is regulated by multiple interconnected metabolic pathways. To comprehensively investigate metabolic alterations under different conditions, we conducted non‐targeted metabolomics analysis followed by KEGG pathway enrichment analysis to identify key metabolic pathways and associated enzymes modulated by HG.

**FIGURE 3:**
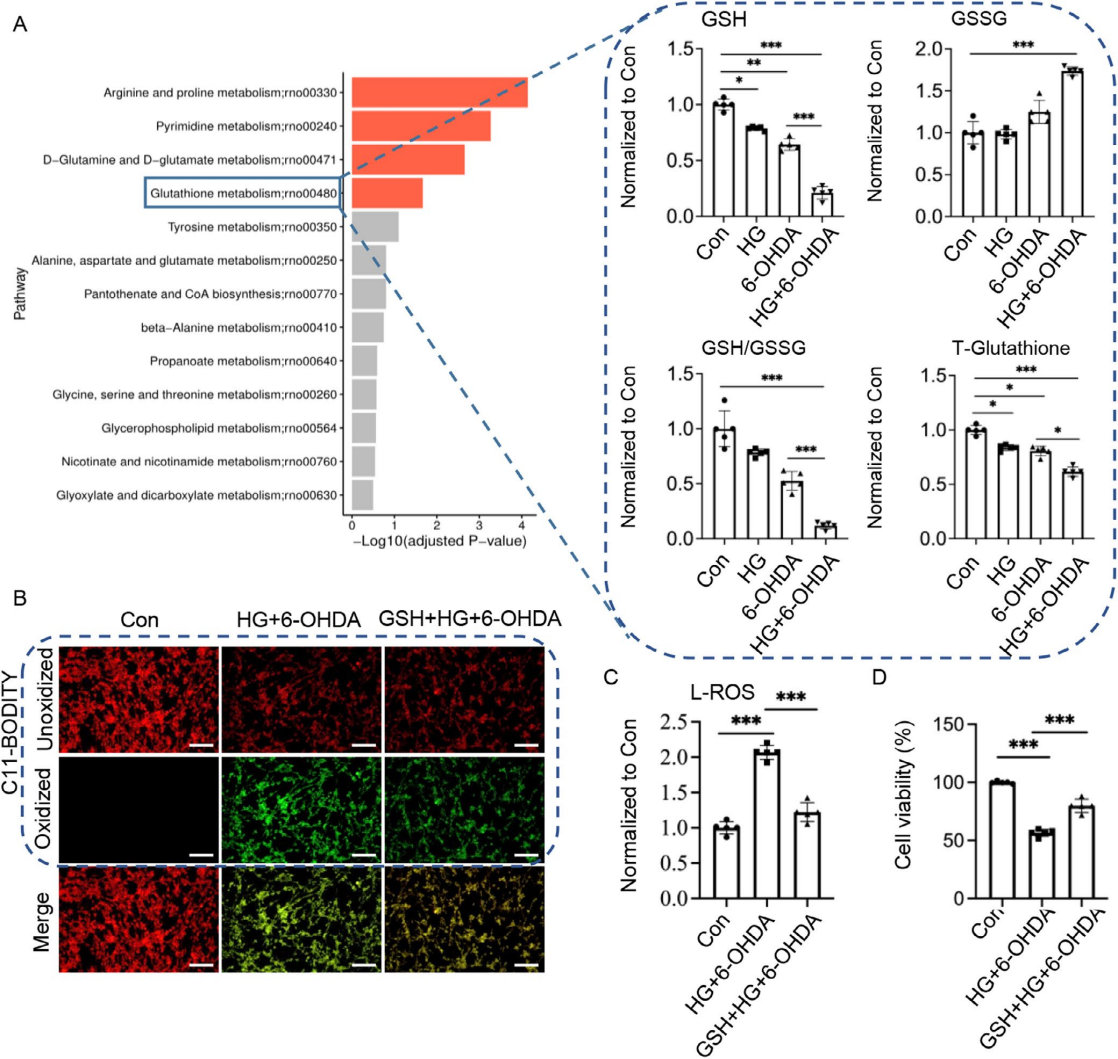
High glucose exacerbates 6‐OHDA‐induced dysregulation of glutathione metabolism. (A) Kyoto Encyclopedia of Genes and Genomes (KEGG) pathway analysis of the top 13 differentially expressed metabolites, highlighting alterations in glutathione metabolism. (B–D) Cells were pretreated with 5 mM GSH for 2 h, followed by HG treatment for 24 h, and then exposed to 50 μM 6‐OHDA for an additional 24 h. (B) Lipid peroxidation was assessed using C11‐BODIPY 581/591. Fluorescent microscopy images show oxidized BODIPY C11 (green) and unoxidized BODIPY C11 (red). Scale bars: 200 μm. (C) Levels of lipid reactive oxygen species (L‐ROS) were quantified using a microplate reader. (D) Cell viability was determined by sulforhodamine B (SRB) assay. Data are presented as mean ± SEM; *n* = 5 biologically independent samples. Statistical analysis was performed using one‐way ANOVA followed by Tukey's multiple comparisons test. *p < 0.05, **p < 0.01, ***p < 0.001.

Our analysis revealed significant dysregulation of glutathione metabolism in 6‐OHDA‐treated PC12 neurons under HG conditions (Figure 3A). While HG alone caused a moderate reduction in reduced glutathione (GSH) and total GSH levels without significantly affecting the GSH/GSSG ratio, 6‐OHDA treatment alone resulted in a substantial decrease in GSH, total GSH, and the GSH/GSSG ratio. These findings suggest that HG primarily impairs GSH synthesis, whereas 6‐OHDA affects both GSH regeneration and synthesis pathways. Notably, the combination of HG and 6‐OHDA synergistically exacerbated GSH depletion, significantly reducing GSH, total GSH, and the GSH/GSSG ratio, indicating that HG potentiates 6‐OHDA‐induced intracellular GSH deficiency by disrupting de novo GSH synthesis.

Supporting this mechanism, exogenous GSH supplementation (ex‐GSH) significantly reduced intracellular lipid peroxide accumulation and attenuated neuronal damage in HG + 6‐OHDA‐treated cells (Figure 3B–D). These results provide compelling evidence that HG facilitates 6‐OHDA‐induced neuronal ferroptosis primarily through inhibition of de novo GSH synthesis, highlighting the critical role of glutathione homeostasis in ferroptosis regulation under hyperglycemic conditions.

### 3.3 HG Inhibited the SLC7A11‐Mediated Cystine Transport, Triggering the Disorder of GSH De Novo Synthesis in 6‐OHDA‐Induced Neurons

**FIGURE 4:**
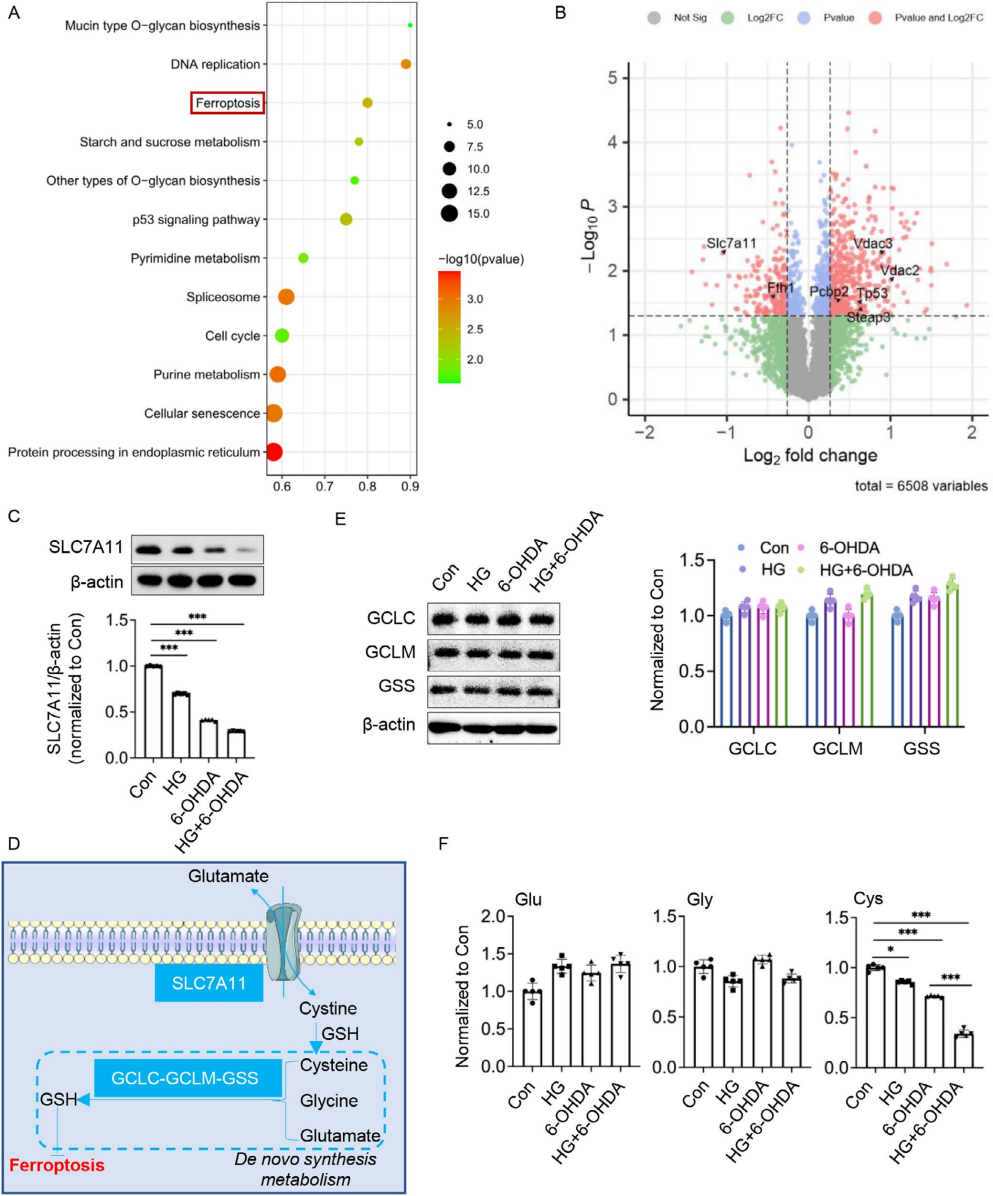
High glucose (HG) impairs SLC7A11‐mediated cystine transport, disrupting de novo glutathione synthesis in 6‐OHDA‐induced neurons. (A) Kyoto Encyclopedia of Genes and Genomes (KEGG) pathway analysis of the top 12 differentially expressed proteins. (B) Volcano plot analysis of differentially expressed proteins. (C) Representative immunoblots of SLC7A11 and β‐Actin in NGF‐differentiated PC12 cells cultured under HG (30 mM glucose) conditions with or without 50 μM 6‐OHDA. (D) Schematic representation of SLC7A11‐mediated cystine transport and its role in de novo synthesis of intracellular glutathione (GSH). (E) Representative immunoblots of GCLC, GCLM, GSS, and β‐Actin in NGF‐differentiated PC12 cells cultured under HG conditions with or without 6‐OHDA. (F) Levels of glutamate (Glu), glycine (Gly), and cysteine (Cys) were quantified using liquid chromatography‐mass spectrometry (LC–MS). Data are presented as mean ± SEM; *n* = 5 biologically independent samples. Statistical analysis was performed using one‐way ANOVA followed by Tukey's multiple comparisons test. *p < 0.05, ***p < 0.001.

To explore the molecular mechanisms by which HG modulates 6‐OHDA‐induced neuronal damage, we conducted quantitative proteomic analysis to compare protein expression profiles between 6‐OHDA‐treated and HG + 6‐OHDA‐treated PC12 neurons. Our proteomic profiling identified significant alterations in the ferroptosis pathway as the most prominently affected pathway under HG conditions (Figure 4A). Notably, we found that SLC7A11 (xCT), a critical component of the cystine/glutamate antiporter system and a key regulator of redox homeostasis, was significantly downregulated in HG + 6‐OHDA‐treated cells compared to controls (Figure 4B). This proteomic observation was further validated by western blot analysis, which confirmed a substantial reduction in SLC7A11 protein levels in 6‐OHDA‐treated PC12 neurons under HG conditions (Figure 4C). The downregulation of SLC7A11, which plays a vital role in maintaining intracellular glutathione levels and protecting against ferroptosis, provides mechanistic insight into how HG exacerbates 6‐OHDA‐induced neuronal damage by disrupting redox homeostasis.

SLC7A11 serves as the core component of the cystine/glutamate antiporter system, playing a pivotal role in cellular defense against ferroptosis by maintaining glutathione (GSH) homeostasis (Figure 4D). This system facilitates the uptake of extracellular cystine, which is subsequently reduced to cysteine—a rate‐limiting precursor for GSH synthesis. Within the cell, cysteine combines with glutamate and glycine through enzymatic reactions catalyzed by glutamate‐cysteine ligase (GCL) and glutathione synthetase (GSS) to generate GSH de novo.

To investigate the molecular mechanisms underlying HG‐induced GSH depletion, we first assessed the expression levels of key enzymes involved in GSH biosynthesis using western blot analysis. Our results showed no significant differences in the protein levels of GCLC, GCLM, and GSS between HG + 6‐OHDA and 6‐OHDA‐treated groups (Figure 4E). Subsequent LC–MS‐based metabolomic analysis of intracellular substrates revealed a specific and significant reduction in cysteine levels, while glutamate and glycine concentrations remained relatively stable (Figure 4F). Notably, the combination of HG and 6‐OHDA treatment resulted in a more pronounced decrease in intracellular cysteine compared to either treatment alone, consistent with the observed downregulation of SLC7A11 protein levels (Figure 4C). These findings suggest that HG‐mediated suppression of SLC7A11 impairs cystine uptake, leading to intracellular cysteine depletion and subsequent disruption of GSH biosynthesis.

Collectively, our data demonstrate that HG exacerbates 6‐OHDA‐induced neuronal vulnerability by compromising the SLC7A11‐mediated cystine/glutamate antiporter system, thereby disrupting cellular redox homeostasis through impaired GSH synthesis. This mechanism provides a molecular basis for understanding how hyperglycemic conditions potentiate ferroptosis in dopaminergic neurons.

### 3.4 Overexpression of SLC7A11 Attenuates HG and 6‐OHDA‐Induced Neuronal Ferroptosis by Suppressing Lipid Peroxidation and Enhancing Cellular Viability

**FIGURE 5:**
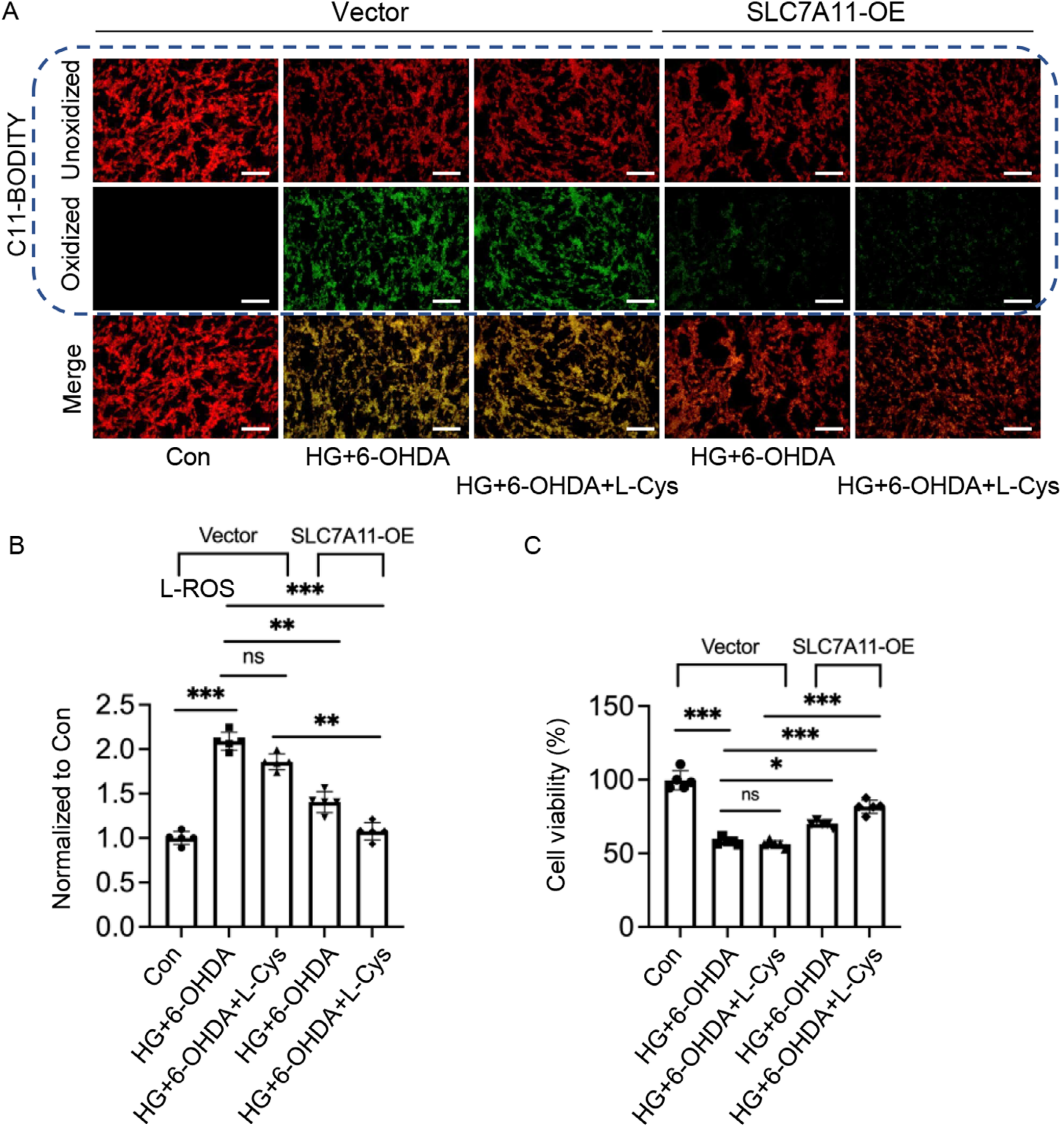
Overexpression of SLC7A11 attenuates lipid peroxidation and cell death in neurons exposed to high glucose and 6‐OHDA. Post‐transfection, cells underwent the following treatment protocol: A 2‐h preconditioning with 0.5 mM cysteine, subsequent 24‐h culture under high glucose conditions, and final 24‐h exposure to 50 μM 6‐OHDA. (A) Lipid peroxidation was assessed using C11‐BODIPY 581/591. Fluorescent microscopy images show oxidized BODIPY C11 (green) and unoxidized BODIPY C11 (red). Scale bars: 200 μm. (B) Levels of lipid reactive oxygen species (L‐ROS) were quantified using a microplate reader. (C) Cell viability was determined by sulforhodamine B (SRB) assay. Data are presented as mean ± SEM; *n* = 5 biologically independent samples. Statistical analysis was performed using one‐way ANOVA followed by Tukey's multiple comparisons test. *p < 0.05, **p < 0.01, ***p < 0.001.

To investigate the protective role of SLC7A11 in HG and 6‐OHDA‐induced ferroptosis, we utilized a gain‐of‐function approach by transiently overexpressing SLC7A11 in PC12 neurons. Our findings reveal that SLC7A11 overexpression (SLC7A11‐OE) significantly mitigates HG + 6‐OHDA‐induced ferroptosis through multiple mechanisms. Quantitative analysis showed that SLC7A11‐OE markedly reduced intracellular lipid peroxidation compared to control cells (Figure 5A,B). Notably, the combination of SLC7A11‐OE with exogenous cystine (L‐Cys) supplementation resulted in a more pronounced reduction in lipid peroxidation, whereas L‐Cys treatment alone had no significant effect, indicating that SLC7A11‐mediated cystine transport is crucial for L‐Cys‐mediated protection.

Additionally, cell viability assays demonstrated that SLC7A11‐OE enhanced neuronal survival under HG + 6‐OHDA conditions, with the combination of SLC7A11‐OE and L‐Cys providing the most robust neuroprotective effect (Figure 5C). These results provide strong evidence that SLC7A11‐mediated cystine transport plays a pivotal role in protecting against HG + 6‐OHDA‐induced neuronal ferroptosis, with the protective effects being contingent on both SLC7A11 expression levels and extracellular cystine availability.

### 3.5 SLC7A11 Overexpression Attenuates 6‐OHDA‐Induced Motor Dysfunction and Nigrostriatal Neurodegeneration in Diabetic Rats

**FIGURE 6:**
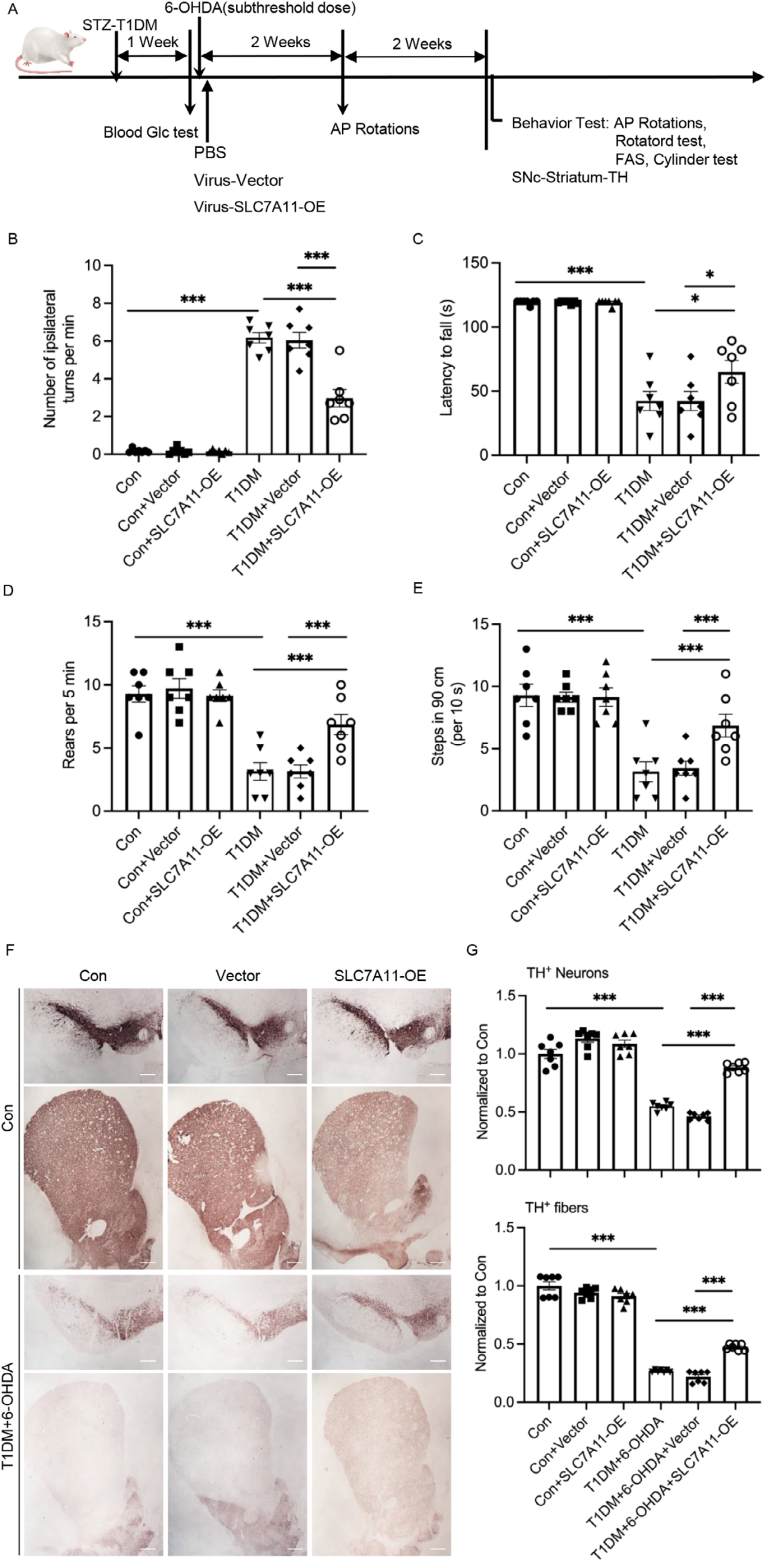
Overexpression of SLC7A11 alleviates motor dysfunction and nigrostriatal neurodegeneration in diabetic rats following 6‐OHDA administration. (A) Schematic representation of the experimental timeline and treatment protocol. (B–E) Behavioral assessments in non‐diabetic or streptozotocin (STZ)‐induced diabetic rats, including (B). AP rotation test, (C) Rotarod test, (D) Cylinder test, and (E) FAS test. (F) Representative immunohistochemical images of coronal sections from the substantia nigra and striatum, stained for tyrosine hydroxylase (TH). Scale bar: 1 mm. (G) Quantification of the proportional stained area of TH immunoreactivity shown in (F). Data are presented as mean ± SEM; *n* = 7 biologically independent animals. Statistical analysis was performed using one‐way ANOVA followed by Tukey's multiple comparisons test. *p < 0.05, ***p < 0.001. Viral constructs: Virus‐Vector: PAAV‐CMV‐mCherry‐3xFLAG‐WPRE Virus‐OE‐SLC7A11: PAAV‐CMV‐mCherry‐P2A‐SLC7A11‐3xFLAG‐WPRE.

To assess the therapeutic potential of SLC7A11 overexpression in mitigating PD progression under diabetic conditions, we performed stereotaxic delivery of pAAV‐CMV‐mCherry‐P2A‐SLC7A11‐3xFLAG‐WPRE vectors into the substantia nigra of diabetic rats following 6‐OHDA administration. After a 4‐week recovery period, comprehensive behavioral assessments were conducted to evaluate motor function (Figure 6A). Strikingly, SLC7A11 overexpression (OE‐SLC7A11) significantly improved motor performance in diabetic rats with 6‐OHDA‐induced parkinsonism, as demonstrated by a battery of behavioral tests. Quantitative analysis revealed that OE‐SLC7A11 treatment enhanced rotarod performance, reduced forelimb asymmetry scores (FAS), increased spontaneous forelimb use in the cylinder test, and decreased amphetamine‐induced rotations compared to 6‐OHDA‐treated diabetic controls (Figure 6B–E).

Histopathological analysis further demonstrated the neuroprotective effects of OE‐SLC7A11, showing increased tyrosine hydroxylase (TH)‐positive neuron counts in the substantia nigra pars compacta (SNc) and elevated TH‐positive fiber density in the striatum compared to 6‐OHDA‐treated diabetic controls (Figure 6F,G). These findings provide robust evidence that SLC7A11 overexpression protects against dopaminergic neurodegeneration and associated motor deficits in diabetic rats with 6‐OHDA‐induced parkinsonism, highlighting its potential as a therapeutic strategy for PD patients with comorbid diabetes.

### 3.6 SLC7A11 Overexpression Ameliorates 6‐OHDA‐Induced Lipid Peroxidation in the Substantia Nigra of Diabetic Rats

**FIGURE 7:**
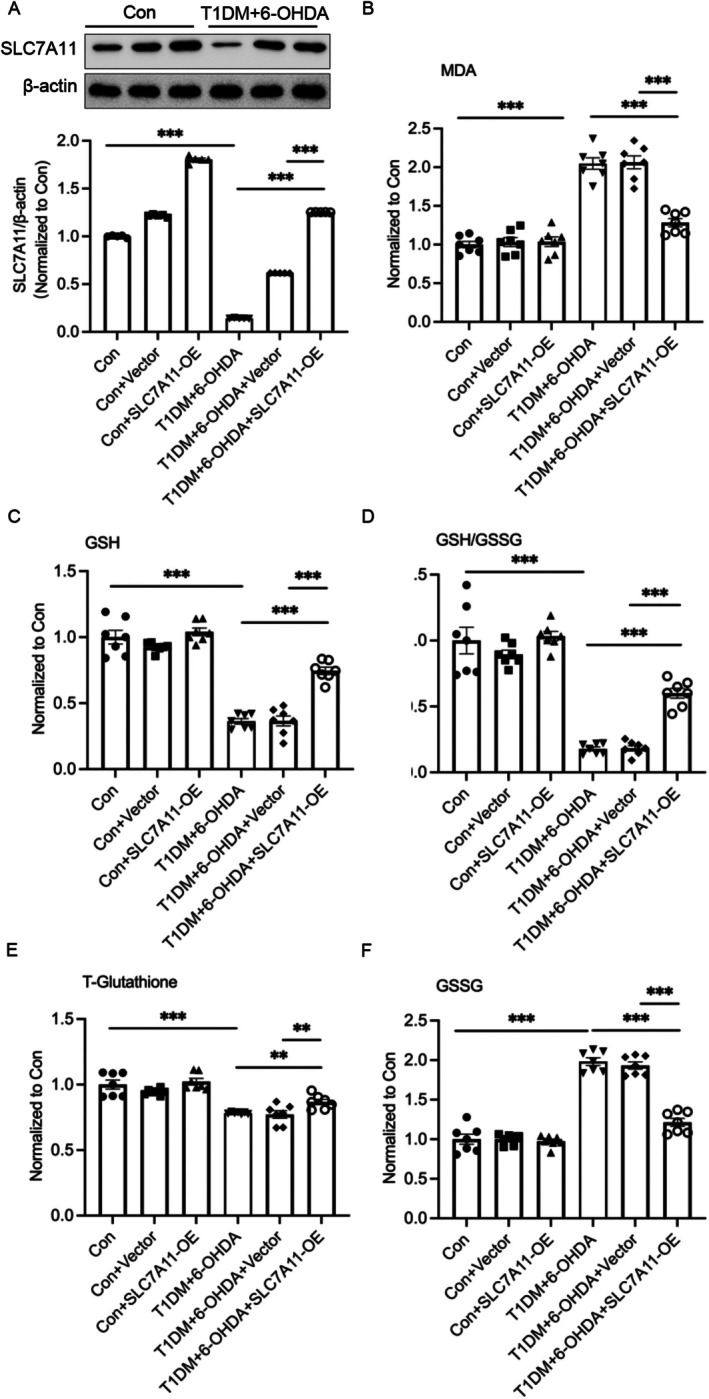
Overexpression of SLC7A11 ameliorates lipid peroxidation in the substantia nigra of diabetic rats following 6‐OHDA administration. The expression of SLC7A11 (A) in the substantia nigra pars compacta (SNc) of non‐diabetic or streptozotocin (STZ)‐induced diabetic rats. (B) Malondialdehyde (MDA) levels in the SNc of non‐diabetic or STZ‐diabetic rats were measured using a Lipid Peroxidation MDA Assay Kit. The level of (C) reduced glutathione (GSH), (D) the GSH/GSSG ratio, (E) total glutathione (T‐Glutathione), and (F) oxidized glutathione (GSSG) in the substantia nigra pars compacta (SNc) of non‐diabetic or streptozotocin (STZ)‐induced diabetic rats were quantified using a GSH and GSSG Assay Kit. Data are presented as mean ± SEM; n=7 biologically independent animals. Statistical analysis was performed using one‐way ANOVA followed by Tukey’s multiple comparisons test. **p < 0.01, ***p < 0.001.

To further explore the antioxidant effects of SLC7A11 overexpression in the substantia nigra (SN) of diabetic PD rats, we quantified lipid peroxidation and glutathione metabolism in this brain region. Using malondialdehyde (MDA) as a sensitive biomarker of lipid peroxidation, we observed a significant increase in MDA levels in T1DM + 6‐OHDA rats compared to controls (Con), which was markedly attenuated by SLC7A11 overexpression (OE‐SLC7A11; Figure 7A,B).

A comprehensive analysis of glutathione metabolism revealed significant alterations in the SN of T1DM + 6‐OHDA rats, including a pronounced reduction in reduced glutathione (GSH), a decreased GSH/GSSG ratio, a decline in total glutathione levels, and an increase in oxidized glutathione (GSSG) compared to controls (Figure 7C–F). Notably, OE‐SLC7A11 treatment effectively restored glutathione homeostasis, increasing GSH levels, the GSH/GSSG ratio, total glutathione, and decreasing GSSG levels compared to T1DM + 6‐OHDA rats without SLC7A11 overexpression.

These findings provide compelling evidence that SLC7A11 plays a critical role in regulating lipid peroxidation and maintaining redox homeostasis in the SN of diabetic rats following 6‐OHDA administration, underscoring its potential as a therapeutic target for PD patients with comorbid diabetes.

### 3.7 SLC7A11 Was Low‐Expressed in Both DM and PD Patients

**FIGURE 8:**
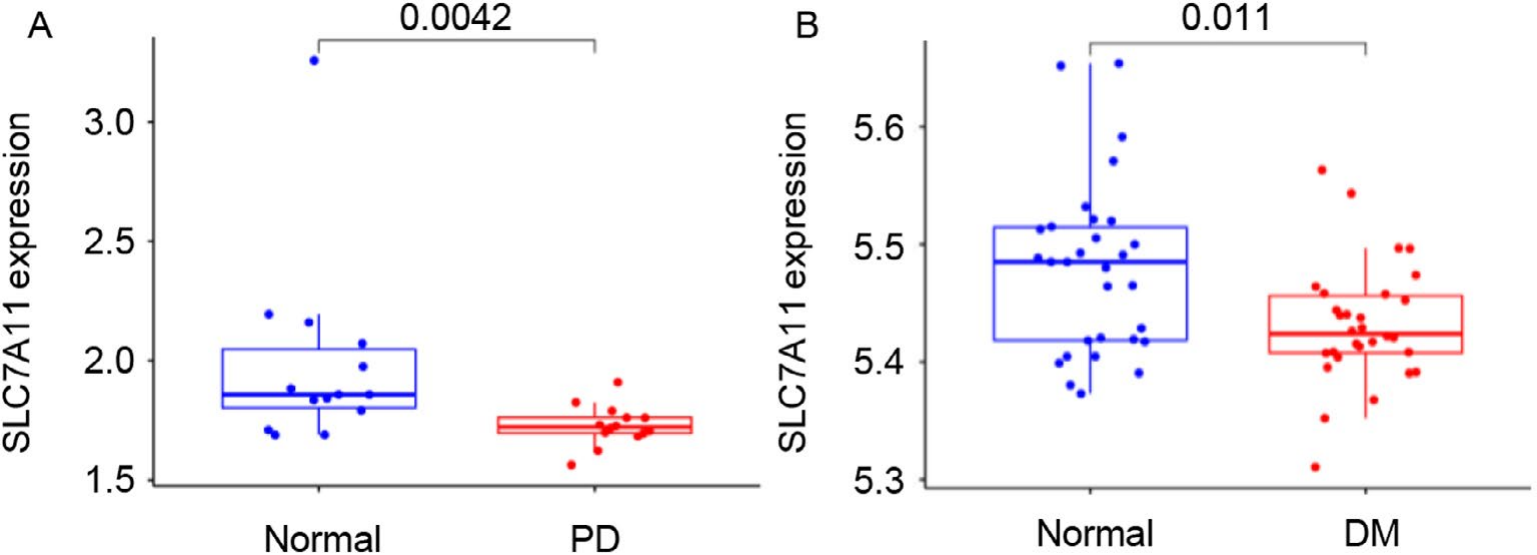
Association between SLC7A11 expression and Parkinson's disease (PD) and diabetes mellitus (DM). (A) Expression levels of SLC7A11 in patients with PD. (B) Expression levels of SLC7A11 in patients with DM.

To elucidate the relationship between SLC7A11 expression and the pathogenesis of DM and PD, we performed an integrated bioinformatics analysis using publicly available transcriptomic datasets. Differential expression analysis revealed a significant downregulation of SLC7A11 in both PD and DM patients compared to healthy controls (Figure 8A,B). These findings suggest that reduced SLC7A11 expression may represent a common molecular signature in both PD and DM, potentially contributing to the increased susceptibility of diabetic patients to PD‐related neurodegeneration through impaired redox homeostasis and increased ferroptosis sensitivity. The consistent downregulation of SLC7A11 across both disease states highlights its potential role as a therapeutic target for neurodegenerative disorders in diabetic populations.

## 4 Discussion

The association between DM and PD has garnered significant attention, with DM identified as a key risk factor for PD, a neurodegenerative disorder characterized by the progressive loss of dopaminergic neurons in the substantia nigra pars compacta and their projections to the striatum. While this correlation is widely recognized, the underlying molecular mechanisms remain incompletely understood. Our study provides novel insights into this relationship by demonstrating that HG conditions in the central nervous system exacerbate 6‐OHDA‐induced ferroptosis through SLC7A11‐mediated mechanisms. Specifically, we revealed that HG disrupts intracellular glutathione (GSH) synthesis by suppressing cystine transport via downregulation of SLC7A11, thereby amplifying 6‐OHDA‐induced ferroptosis. This pathological cascade was effectively reversed by SLC7A11 overexpression, which restored redox homeostasis and attenuated ferroptosis. Importantly, therapeutic upregulation of SLC7A11 expression significantly improved motor function and prevented nigrostriatal dopaminergic neurodegeneration in diabetic PD rat models. These findings establish a mechanistic link between DM and PD pathogenesis, demonstrating that HG‐induced SLC7A11 deficiency disrupts glutathione metabolism and promotes ferroptosis, ultimately contributing to dopaminergic neuron loss.

### 4.1 Hyperglycemia as a Risk Factor for PD Pathogenesis

DM, characterized by chronic hyperglycemia, is a well‐established contributor to the increased risk of PD. Clinical studies have shown that elevated hemoglobin A1c (HbA1c) levels (≥ 7%) and advanced Hoehn‐Yahr (HeY) staging independently predict cognitive decline in PD patients, while dysregulated blood glucose levels are strongly associated with cognitive impairment in PD [29, 30]. Our previous work demonstrated that diabetic hyperglycemia exacerbates motor deficits and enhances the vulnerability of dopaminergic neurons to the neurotoxic effects of 6‐OHDA in rodent models of PD [26]. These findings underscore the role of HG in accelerating neuronal degeneration, although the precise mechanisms remain elusive. Recent epidemiological and experimental studies have revealed shared pathophysiological mechanisms between type 2 diabetes (T2D) and PD, including oxidative stress, chronic inflammation, and ferroptosis [31]. Chronic inflammation exacerbates oxidative stress by promoting reactive oxygen species (ROS) generation through immune cell activation and inflammatory signaling pathways. Conversely, oxidative stress perpetuates inflammation by triggering downstream pathways that enhance pro‐inflammatory cytokine production, establishing a self‐amplifying vicious cycle. Notably, ROS accumulation—especially in an iron‐rich microenvironment—drives lipid peroxidation, a defining feature of ferroptosis. Furthermore, inflammation modulates ferroptosis by dysregulating iron metabolism and ROS synthesis. Pro‐inflammatory cytokines disrupt iron homeostasis, elevating labile iron pools that catalyze lipid peroxidation and accelerate ferroptotic cell death [32]. In this study, we identified that HG elevates intracellular lipid peroxide levels and potentiates 6‐OHDA‐induced ferroptosis in neurons (Figure 2). This process was effectively reversed by ferrostatin‐1 (Fer‐1), a synthetic antioxidant that inhibits lipid peroxidation and prevents ferroptosis (Figure S1). These observations suggest that ferroptosis is a key mechanism through which HG promotes neuronal degeneration, highlighting the importance of investigating HG‐induced ferroptosis in the context of PD complicating DM.

### 4.2 Ferroptosis in PD Pathogenesis

Ferroptosis, an iron‐dependent form of regulated cell death, plays a critical role in the demise of dopaminergic neurons in PD [28]. This aligns with the pathological hallmarks of PD, including elevated iron levels [33], accumulation of lipid peroxides [34, 35, 36], and deficiencies in antioxidant defense systems, such as reduced levels of SLC7A11 [37], DJ‐1 [38], GSH [39], and coenzyme Q10 [40]. Our findings demonstrate that HG primarily disrupts the antioxidant system in neurons exposed to 6‐OHDA toxicity. Through integrated non‐targeted metabolomics and proteomics analyses, we revealed that HG suppresses SLC7A11‐mediated cystine transport, leading to impaired intracellular GSH synthesis and exacerbating 6‐OHDA‐induced ferroptosis (Figures 3 and 4). Furthermore, analysis of gene expression profiles from the GEO database revealed downregulated SLC7A11 expression in both PD and DM patients, underscoring the pivotal role of SLC7A11 deficiency in amplifying PD risk in diabetic individuals.

### 4.3 SLC7A11 as a Central Regulator of Ferroptosis

SLC7A11, a key component of the cystine/glutamate antiporter system, is a critical regulator of ferroptosis. Its downregulation disrupts GSH synthesis and promotes lipid peroxide accumulation by impairing cystine uptake [41, 42]. Notably, the ferroptosis inducer erastin specifically targets SLC7A11 to trigger cell death [43, 44], while the tumor suppressor p53 represses SLC7A11 transcription, further disrupting GSH synthesis and promoting ferroptosis [45, 46]. Our findings demonstrate that upregulation of the cystine/SLC7A11/GSH axis effectively counteracts HG + 6‐OHDA‐induced ferroptosis. Both exogenous GSH supplementation and SLC7A11 overexpression (OE‐SLC7A11) significantly mitigated neuronal ferroptosis under HG + 6‐OHDA conditions. Importantly, the combination of cystine and OE‐SLC7A11 provided more robust protection than OE‐SLC7A11 alone, highlighting the dependence of HG's effects on the cystine/SLC7A11/GSH system in 6‐OHDA‐induced ferroptosis. Furthermore, OE‐SLC7A11 improved motor deficits, reduced lipid peroxidation, and attenuated neurodegeneration in the nigrostriatal region of diabetic rats following 6‐OHDA treatment.

## 5 Conclusion

Our study elucidates a critical link between the hyperglycemic environment induced by diabetes and the increased susceptibility to PD. The findings demonstrate that this heightened vulnerability is mediated through the suppression of the cystine/SLC7A11/glutathione (GSH) axis, which exacerbates neuronal sensitivity to PD‐inducing agents such as 6‐OHDA, ultimately driving neuronal ferroptosis. Importantly, we have shown that upregulation of SLC7A11 expression can effectively counteract this increased PD risk in diabetic conditions. These results provide novel mechanistic insights into the pathophysiological connection between diabetes‐associated hyperglycemia and PD development, while simultaneously identifying SLC7A11 as a promising therapeutic target for the clinical management of PD in diabetic populations. The implications of these findings extend beyond the immediate context, potentially informing future therapeutic strategies for neurodegenerative disorders in metabolic disease settings.

## Author Contributions

**Ya Zhao:** conceptualization, data curation, funding acquisition, methodology, project administration, resources, writing – original draft, and writing – review and editing. **Dan Wang:** data curation, formal analysis, funding acquisition, investigation, and methodology. **Yanwei Wang:** investigation, software, and validation. **Dan Mu:** visualization and writing – review and editing. **Lang Qu:** funding acquisition, resources, supervision, and writing – review and editing. **Rong Li:** conceptualization, funding acquisition, project administration, resources, supervision, and writing – review and editing.

## Ethics Statement

The permission of animal use were approved by the Institutional Animal Care and Use Committee of North Sichuan Medical College (approval number NSMC 2024108) and conducted in accordance with ARRIVE guidelines 2.0 and the NIH Guide for the Care and Use of Laboratory Animals.

## Conflicts of Interest

The authors declare no conflicts of interest.

## Supporting information


Appendix S1:

## Data Availability

All data provided in this paper are available from the corresponding author upon reasonable request.

## References

[1] E. De Pablo‐Fernandez , R. Goldacre , J. Pakpoor , A. J. Noyce , and T. T. Warner , “Association Between Diabetes and Subsequent Parkinson Disease: A Record‐Linkage Cohort Study,” Neurology 91 (2018): e139–e142. 29898968 10.1212/WNL.0000000000005771

[2] E. De Pablo‐Fernandez , F. Sierra‐Hidalgo , J. Benito‐León , and F. Bermejo‐Pareja , “Association Between Parkinson's Disease and Diabetes: Data From NEDICES Study,” Acta Neurologica Scandinavica 136 (2017): 732–736. 28653373 10.1111/ane.12793

[3] S. M. Jeong , K. Han , D. Kim , S. Y. Rhee , W. Jang , and D. W. Shin , “Body Mass Index, Diabetes, and the Risk of Parkinson's Disease,” Movement Disorders: Official Journal of the Movement Disorder Society 35 (2020): 236–244. 31785021 10.1002/mds.27922

[4] X. Yue , H. Li , H. Yan , P. Zhang , L. Chang , and T. Li , “Risk of Parkinson Disease in Diabetes Mellitus: An Updated Meta‐Analysis of Population‐Based Cohort Studies,” Medicine 95 (2016): e3549. 27149468 10.1097/MD.0000000000003549 PMC4863785

[5] D. Bosco , M. Plastino , D. Cristiano , et al., “Dementia Is Associated With Insulin Resistance in Patients With Parkinson's Disease,” Journal of the Neurological Sciences 315 (2012): 39–43. 22265943 10.1016/j.jns.2011.12.008

[6] J. K. Morris , E. D. Vidoni , R. D. Perea , et al., “Insulin Resistance and Gray Matter Volume in Neurodegenerative Disease,” Neuroscience 270 (2014): 139–147. 24735819 10.1016/j.neuroscience.2014.04.006 PMC4211112

[7] H. Chohan , K. Senkevich , R. K. Patel , et al., “Type 2 Diabetes as a Determinant of Parkinson's Disease Risk and Progression,” Movement Disorders: Official Journal of the Movement Disorder Society 36 (2021): 1420–1429. 33682937 10.1002/mds.28551 PMC9017318

[8] K. Kawabe , K. Ikeda , and Y. Iwasaki , “Clinical Features of Parkinson Disease When Onset of Diabetes Came First: A Case‐Control Study,” Neurology 79 (2012): 1835–1836. 10.1212/WNL.0b013e3182742edb 23091078

[9] V. Kotagal , R. L. Albin , M. L. T. M. Müller , R. A. Koeppe , K. A. Frey , and N. I. Bohnen , “Diabetes Is Associated With Postural Instability and Gait Difficulty in Parkinson Disease,” Parkinsonism & Related Disorders 19 (2013): 522–526. 23462483 10.1016/j.parkreldis.2013.01.016 PMC3607954

[10] N. I. Bohnen , V. Kotagal , M. L. T. M. Müller , et al., “Diabetes Mellitus Is Independently Associated With More Severe Cognitive Impairment in Parkinson Disease,” Parkinsonism & Related Disorders 20 (2014): 1394–1398. 25454317 10.1016/j.parkreldis.2014.10.008 PMC4314515

[11] M. Petrou , C. Davatzikos , M. Hsieh , et al., “Diabetes, Gray Matter Loss, and Cognition in the Setting of Parkinson Disease,” Academic Radiology 23 (2016): 577–581. 26874576 10.1016/j.acra.2015.07.014 PMC4859345

[12] H. Wang , “MicroRNAs, Parkinson's Disease, and Diabetes Mellitus,” International Journal of Molecular Sciences 22 (2021): 2953. 33799467 10.3390/ijms22062953 PMC8001823

[13] S. J. Chung , S. Jeon , H. S. Yoo , et al., “Detrimental Effect of Type 2 Diabetes Mellitus in a Large Case Series of Parkinson's Disease,” Parkinsonism & Related Disorders 64 (2019): 54–59. 30193817 10.1016/j.parkreldis.2018.08.023

[14] I. Pérez‐Taboada , S. Alberquilla , E. D. Martín , et al., “Diabetes Causes Dysfunctional Dopamine Neurotransmission Favoring Nigrostriatal Degeneration in Mice,” Movement Disorders: Official Journal of the Movement Disorder Society 35 (2020): 1636–1648. 32666590 10.1002/mds.28124 PMC7818508

[15] D. Sergi , J. Renaud , N. Simola , and M. G. Martinoli , “Diabetes, a Contemporary Risk for Parkinson's Disease: Epidemiological and Cellular Evidences,” Frontiers in Aging Neuroscience 11 (2019): 302. 31787891 10.3389/fnagi.2019.00302 PMC6856011

[16] O. M. Ighodaro , “Molecular Pathways Associated With Oxidative Stress in Diabetes Mellitus,” Biomedicine & Pharmacotherapy = Biomedecine & Pharmacotherapie 108 (2018): 656–662. 30245465 10.1016/j.biopha.2018.09.058

[17] N. K. Mule and J. N. Singh , “Diabetes Mellitus to Neurodegenerative Disorders: Is Oxidative Stress Fueling the Flame?,” CNS & Neurological Disorders Drug Targets 17 (2018): 644–653. 30091419 10.2174/1871527317666180809092359

[18] J. Renaud , V. Bassareo , J. Beaulieu , et al., “Dopaminergic Neurodegeneration in a Rat Model of Long‐Term Hyperglycemia: Preferential Degeneration of the Nigrostriatal Motor Pathway,” Neurobiology of Aging 69 (2018): 117–128. 29890391 10.1016/j.neurobiolaging.2018.05.010

[19] P. Salahuddin , G. Rabbani , and R. H. Khan , “The Role of Advanced Glycation End Products in Various Types of Neurodegenerative Disease: A Therapeutic Approach,” Cellular & Molecular Biology Letters 19 (2014): 407–437. 25141979 10.2478/s11658-014-0205-5 PMC6275793

[20] H. Vicente Miranda , É. M. Szegő , L. M. A. Oliveira , et al., “Glycation Potentiates α‐Synuclein‐Associated Neurodegeneration in Synucleinopathies,” Brain: A Journal of Neurology 140 (2017): 1399–1419. 28398476 10.1093/brain/awx056

[21] L. Wang , Y. Q. Zhai , L. L. Xu , et al., “Metabolic Inflammation Exacerbates Dopaminergic Neuronal Degeneration in Response to Acute MPTP Challenge in Type 2 Diabetes Mice,” Experimental Neurology 251 (2014): 22–29. 24220636 10.1016/j.expneurol.2013.11.001

[22] S. A. Liddelow , K. A. Guttenplan , L. E. Clarke , et al., “Neurotoxic Reactive Astrocytes Are Induced by Activated Microglia,” Nature 541 (2017): 481–487. 28099414 10.1038/nature21029 PMC5404890

[23] Y. Deng , Y. Zhang , Y. Li , et al., “Occurrence and Distribution of Salsolinol‐Like Compound, 1‐Acetyl‐6,7‐Dihydroxy‐1,2,3,4‐Tetrahydroisoquinoline (ADTIQ) in Parkinsonian Brains,” Journal of Neural Transmission 119 (2012): 435–441. 22065205 10.1007/s00702-011-0724-4

[24] B. Xie , F. Lin , K. Ullah , et al., “A Newly Discovered Neurotoxin ADTIQ Associated With Hyperglycemia and Parkinson's Disease,” Biochemical and Biophysical Research Communications 459 (2015): 361–366. 25744031 10.1016/j.bbrc.2015.02.069

[25] R. Brauer , L. Wei , T. Ma , et al., “Diabetes Medications and Risk of Parkinson's Disease: A Cohort Study of Patients With Diabetes,” Brain: A Journal of Neurology 143 (2020): 3067–3076. 33011770 10.1093/brain/awaa262 PMC7794498

[26] Y. Zhao , Y. Wang , Y. Wu , et al., “PKM2‐Mediated Neuronal Hyperglycolysis Enhances the Risk of Parkinson's Disease in Diabetic Rats,” Journal of Pharmaceutical Analysis 13 (2023): 187–200. 36908857 10.1016/j.jpha.2022.11.006 PMC9999299

[27] Y. Sun , L. He , W. Wang , et al., “Activation of Atg7‐Dependent Autophagy by a Novel Inhibitor of the Keap1‐Nrf2 Protein‐Protein Interaction From *Penthorum chinense* Pursh. Attenuates 6‐Hydroxydopamine‐Induced Ferroptosis in Zebrafish and Dopaminergic Neurons,” Food & Function 13 (2022): 7885–7900. 35776077 10.1039/d2fo00357k

[28] B. Do Van , F. Gouel , A. Jonneaux , et al., “Ferroptosis, a Newly Characterized Form of Cell Death in Parkinson's Disease That Is Regulated by PKC,” Neurobiology of Disease 94 (2016): 169–178. 27189756 10.1016/j.nbd.2016.05.011

[29] S. Luo , B. C. Angelo , T. Chow , et al., “Associations Between Exposure to Gestational Diabetes Mellitus in Utero and Daily Energy Intake, Brain Responses to Food Cues, and Adiposity in Children,” Diabetes Care 44 (2021): 1185–1193. 33827804 10.2337/dc20-3006 PMC8132328

[30] L. Yang , Z. Chen , B. Li , et al., “Multiple Evidences for Association Between Cognitive Impairment and Dysglycemia in Parkinson's Disease: Implications for Clinical Practice,” Frontiers in Aging Neuroscience 9 (2017): 355. 29163137 10.3389/fnagi.2017.00355 PMC5675878

[31] I. Stoian , “Ferroptosis—A Shared Mechanism for Parkinson's Disease and Type 2 Diabetes,” International Journal of Molecular Sciences 25 (2024): 8838. 39201524 10.3390/ijms25168838 PMC11354749

[32] I. Stoian , “Targeting Ferroptosis in Parkinson's: Repurposing Diabetes Drugs as a Promising Treatment,” International Journal of Molecular Sciences 26 (2025): 1516. 40003982 10.3390/ijms26041516 PMC11855881

[33] F. Depierreux , E. Parmentier , L. Mackels , et al., “Parkinson's Disease Multimodal Imaging: F‐DOPA PET, Neuromelanin‐Sensitive and Quantitative Iron‐Sensitive MRI,” NPJ Parkinson's Disease 7 (2021): 57. 10.1038/s41531-021-00199-2 PMC8266835 34238927

[34] W. Y. Sun , V. A. Tyurin , K. Mikulska‐Ruminska , et al., “Phospholipase iPLA(2)β Averts Ferroptosis by Eliminating a Redox Lipid Death Signal,” Nature Chemical Biology 17 (2021): 465–476. 33542532 10.1038/s41589-020-00734-x PMC8152680

[35] C. C. de Farias , M. Maes , K. L. Bonifácio , et al., “Highly Specific Changes in Antioxidant Levels and Lipid Peroxidation in Parkinson's Disease and Its Progression: Disease and Staging Biomarkers and New Drug Targets,” Neuroscience Letters 617 (2016): 66–71. 26861200 10.1016/j.neulet.2016.02.011

[36] C. Vida , H. Kobayashi , A. Garrido , et al., “Lymphoproliferation Impairment and Oxidative Stress in Blood Cells From Early Parkinson's Disease Patients,” International Journal of Molecular Sciences 20 (2019): 771. 30759742 10.3390/ijms20030771 PMC6386872

[37] C. L. Vallerga , F. Zhang , J. Fowdar , et al., “Analysis of DNA Methylation Associates the Cystine‐Glutamate Antiporter SLC7A11 With Risk of Parkinson's Disease,” Nature Communications 11 (2020): 1238. 10.1038/s41467-020-15065-7 PMC7060318 32144264

[38] J. Cao , X. Chen , L. Jiang , et al., “DJ‐1 Suppresses Ferroptosis Through Preserving the Activity of S‐Adenosyl Homocysteine Hydrolase,” Nature Communications 11 (2020): 1251. 10.1038/s41467-020-15109-y PMC7060199 32144268

[39] C. Venkateshappa , G. Harish , R. B. Mythri , A. Mahadevan , M. M. Srinivas Bharath , and S. K. Shankar , “Increased Oxidative Damage and Decreased Antioxidant Function in Aging Human Substantia Nigra Compared to Striatum: Implications for Parkinson's Disease,” Neurochemical Research 37 (2012): 358–369. 21971758 10.1007/s11064-011-0619-7

[40] L. K. Mischley , J. Allen , and R. Bradley , “Coenzyme Q10 Deficiency in Patients With Parkinson's Disease,” Journal of the Neurological Sciences 318 (2012): 72–75. 22542608 10.1016/j.jns.2012.03.023 PMC3366011

[41] P. Koppula , L. Zhuang , and B. Gan , “Cystine Transporter SLC7A11/xCT in Cancer: Ferroptosis, Nutrient Dependency, and Cancer Therapy,” Protein & Cell 12 (2021): 599–620. 33000412 10.1007/s13238-020-00789-5 PMC8310547

[42] Y. Iida , M. Okamoto‐Katsuyama , S. Maruoka , et al., “Effective Ferroptotic Small‐Cell Lung Cancer Cell Death From SLC7A11 Inhibition by Sulforaphane,” Oncology Letters 21 (2021): 71. 33365082 10.3892/ol.2020.12332 PMC7716721

[43] X. Xu , X. Zhang , C. Wei , et al., “Targeting SLC7A11 Specifically Suppresses the Progression of Colorectal Cancer Stem Cells via Inducing Ferroptosis,” European Journal of Pharmaceutical Sciences: Official Journal of the European Federation for Pharmaceutical Sciences 152 (2020): 105450. 32621966 10.1016/j.ejps.2020.105450

[44] L. Chen , L. Qiao , Y. Bian , and X. Sun , “GDF15 Knockdown Promotes Erastin‐Induced Ferroptosis by Decreasing SLC7A11 Expression,” Biochemical and Biophysical Research Communications 526 (2020): 293–299. 32209255 10.1016/j.bbrc.2020.03.079

[45] Z. Guan , J. Chen , X. Li , and N. Dong , “Tanshinone IIA Induces Ferroptosis in Gastric Cancer Cells Through p53‐Mediated SLC7A11 Down‐Regulation,” Bioscience Reports 40 (2020). 10.1042/BSR20201807 PMC7953492 32776119

[46] Y. Luo , X. Gao , L. Zou , M. Lei , J. Feng , and Z. Hu , “Bavachin Induces Ferroptosis Through the STAT3/P53/SLC7A11 Axis in Osteosarcoma Cells,” Oxidative Medicine and Cellular Longevity 2021 (2021): 1783485. 34707773 10.1155/2021/1783485 PMC8545544

