# Supplementary material for: Hyperglycemia Aggravates 6‐Hydroxydopamine‐Induced Neuronal Ferroptosis via SLC7A11‐Dependent Pathway in Diabetic PD Rat Model
Source: CNS Neurosci Ther. 2025 Jul 4;31(7):e70487. doi: 10.1111/cns.70487 (PMC12231211; doi:10.1111/cns.70487)
Supplement: Supplementary file 1 — Appendix S1: [file CNS-31-e70487-s001.docx]

**Supporting information**

**High glucose exacerbates 6-OHDA-induced neuronal ferroptosis via a SLC7A11 dependent manner in diabetic PD rats**

**Ya Zhao^†1^, Dan Wang^†3^,Yanwei Wang^4^, Dan Mu^6^, Lang Qu^*5^, Rong Li^1,2*^**

This supporting information includes three Texts and two figures.

Text S1. Metabolite extraction, profiling, and identification…………………………………2

Text S2. Proteome sample analysis and quantitation………………………………………3

Text S3 Behavioral Assessments………………………………………………………………4

Supplementary Figure 1. ………………………………………………………………………5

Supplementary Figure 2. ………………………………………………………………………6

Text S1. Metabolite Extraction, Profiling, and Identification

Cell samples were homogenized in a suspension buffer containing 0.25 M Tris sucrose EDTA (pH 7.4) and 0.1% sodium lauryl sulfate. Metabolites were extracted using a solvent mixture of water, methanol, and chloroform in a 1:1.5:2 (v/v) ratio. The organic phase was collected, evaporated under a gentle stream of nitrogen gas, and reconstituted in 0.5 mL of methanol. Particulate matter was removed by filtration through a 0.45 μm Acrodisc syringe filter (25 mm diameter). The resulting filtrate was stored in the dark at 4°C until further analysis.

Metabolite profiling was performed using a ThermoFisher Scientific Vanquish Ultra-High Performance Liquid Chromatography (UHPLC) system coupled to a Q-Exactive Orbitrap mass spectrometer. Chromatographic separation was achieved on a Waters UPLC BEH amide column (1.7 µm particle size, 100 mm × 2.1 mm).

For negative ionization mode, the mobile phase consisted of solvent A (25 mM ammonium hydroxide and 25 mM ammonium acetate in water) and solvent B (acetonitrile). The gradient elution program was as follows: 0–0.5 min, 95% B; 0.5–9 min, 95% to 65% B; 9–10 min, 65% to 40% B; 10–12 min, 40% B; 12–12.1 min, 40% to 95% B; 12.1–15 min, 95% B, with a constant flow rate of 0.4 mL/min. For positive ionization mode, solvent A was water containing 0.1% formic acid, and solvent B was acetonitrile. The gradient program was: 0–1 min, 5% B; 1–10 min, 5% to 100% B; 10–12 min, 100% B; 12–12.1 min, 100% to 5% B; 12.1–15 min, 5% B, at a flow rate of 0.4 mL/min.

Mass spectrometry detection was performed in both positive and negative ionization modes with ion spray voltages set at 4.5 kV and -3.0 kV, respectively. The interface, heat block, and desolvation line temperatures were maintained at 300°C, 400°C, and 250°C, respectively. Drying and nebulizing gas flow rates were set to 10 L/min and 3 L/min, respectively. Full-scan mass spectra were acquired over a range of m/z 70–1050 Da in both ionization modes, with collision energies of 10, 20, and 40 eV applied for fragmentation.

Data acquisition and pre-analytical processing were conducted using Xcalibur Software v.4.1 (Thermo Fisher Scientific Inc.). Quality control samples were analyzed to ensure data reliability, and peaks with a coefficient of variation (CV) exceeding 30% were excluded from further analysis. Multivariate statistical analysis was performed using R software. Metabolite identification was achieved by matching MS/MS spectra against the METLIN (<http://metlin.scripps.edu/>) and Human Metabolome Database (HMDB; <http://www.hmdb.ca/>) repositories. Pathway enrichment analysis was conducted using the Kyoto Encyclopedia of Genes and Genomes (KEGG) database ([www.genome.jp/kegg/](http://www.genome.jp/kegg/)).

Text S2. Proteome sample analysis and quantitation.

For proteomic analysis, peptide samples were prepared for mass spectrometry (MS) using a standardized digestion protocol. Briefly, 25 µL of cell lysate was mixed with 20 mM tris(2-carboxyethyl)phosphine (TCEP), 25% trifluoroethanol (TFE), and 35 µL of 100 mM triethylammonium bicarbonate (TEAB) buffer (pH 8.5), followed by incubation at 55°C for 20 minutes to facilitate reduction. Alkylation was then performed by adding 55 mM 2-chloroacetamide (CAA) to the mixture, which was incubated in the dark for 30 minutes. Subsequently, 2.5 µg of trypsin was added, and the samples were digested overnight at 37°C. After digestion, the samples were centrifuged at 20,000 × g for 10 minutes, and the supernatant was dried and reconstituted in 50 µL of 100 mM TEAB (pH 8.5).

For tandem mass tag (TMT) labeling, 25 µg of peptides from each sample were labeled with TMT reagents and incubated overnight at room temperature. The labeled peptides were pooled and purified using a C18 solid-phase extraction (SPE) column, followed by fractionation on a C18 rotating column. Peptide fractions (2 µg each) were separated using a 50 cm × 75 μm Easy Spray column (Thermo Scientific) on a Proxeon 1000 UHPLC system coupled to an Orbitrap Exploris 480 mass spectrometer (Thermo Scientific). Chromatographic separation was achieved over a 90-minute gradient using mobile phase A (0.1% formic acid in water) and mobile phase B (0.1% formic acid in 99% acetonitrile).

MS data were acquired in data-dependent acquisition (DDA) mode, with full-scan MS spectra collected at a resolution of 75,000 and MS/MS spectra at a resolution of 50,000. Raw data were processed using Proteome Discoverer 2.1 software (Thermo Scientific) and Mascot 2.6.0 (Matrix Science) for protein identification and quantification. Search parameters included a precursor mass tolerance of 20 ppm, a fragment mass tolerance of 0.04 Da, trypsin/P as the proteolytic enzyme, and a maximum of three missed cleavages. Dynamic modifications accounted for N-terminal acetylation, methionine oxidation, and asparagine/glutamine deamidation, while static modifications included carbamidomethylation of cysteine and TMT10-plex labeling of lysine residues and peptide N-termini.

To ensure high-confidence identifications, decoy database searches were performed with strict and relaxed false discovery rates (FDR) set at 1% and 5%, respectively. Peptide sequences were matched against a local mouse proteome database from Uniprot. Protein quantification was based on unique and razor peptides, with TMT reporter ion intensities normalized for downstream statistical analysis. Ribosomal proteins (RPL and RPS) were excluded from the analysis to focus on high-confidence target proteins.

Text S3. Behavioral assessments

Behavioral tests were conducted 4 weeks post-6-OHDA injection between 9:00 AM and 5:00 PM by experimenters blinded to treatment groups:

Rotarod Test

Motor coordination was assessed using an accelerating rotarod (Model 47700, Ugo Basile) with speed increasing from 5 to 20 rpm over 2 minutes. Rats were acclimated to the apparatus for 3 consecutive days prior to testing. Three trials were conducted with 10-minute inter-trial intervals, and the latency to fall was recorded.

Forepaw Adjusting Steps (FAS) Test

Forelimb akinesia was evaluated by gently holding the rat's hindlimbs and one forelimb while allowing the other forelimb to contact a smooth table surface. The rat was moved laterally at a constant speed of 90 cm/10 s for six trials (three in each direction) with 10-second intervals. Adjusting steps were counted by two independent observers.

Cylinder Test

Spontaneous forelimb use was assessed by placing rats individually in a transparent acrylic cylinder (20 cm diameter × 30 cm height) for 5 minutes(30). Sessions were recorded using a high-definition camera (Sony FDR-AX100) and analyzed offline by counting ipsilateral and contralateral forepaw contacts relative to the 6-OHDA lesion side.

Amphetamine-Induced Rotation

Rotational behavior was quantified for 90 minutes following intraperitoneal injection of D-amphetamine sulfate (5 mg/kg; Sigma-Aldrich). The number of complete body rotations towards the side where the 6-OHDA lesion was made was recorded.


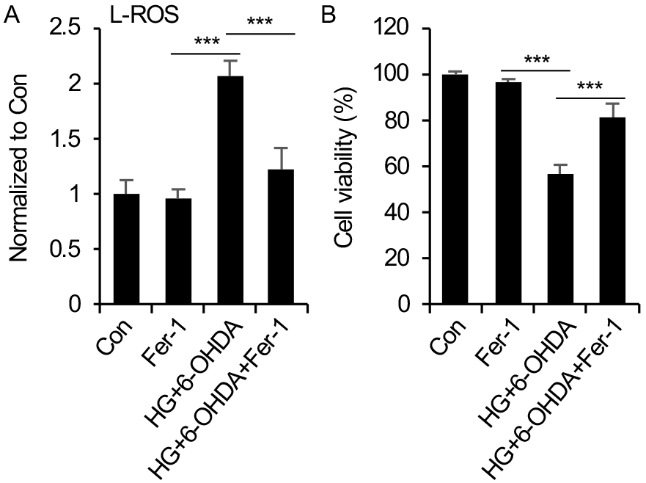


Supplementary Figure 1. Ferrostatin-1 (Fer-1) attenuates lipid reactive oxygen species (L-ROS) accumulation and cell damage in PC12 cells exposed to high glucose (HG) and 6-OHDA. (A) Levels of L-ROS were quantified using a microplate reader. (B) Cell viability was assessed by sulforhodamine B (SRB) assay. Data are presented as mean ± SEM; n = 3 biologically independent samples. Statistical analysis was performed using one-way ANOVA followed by Tukey’s multiple comparisons test. ***P < 0.001.


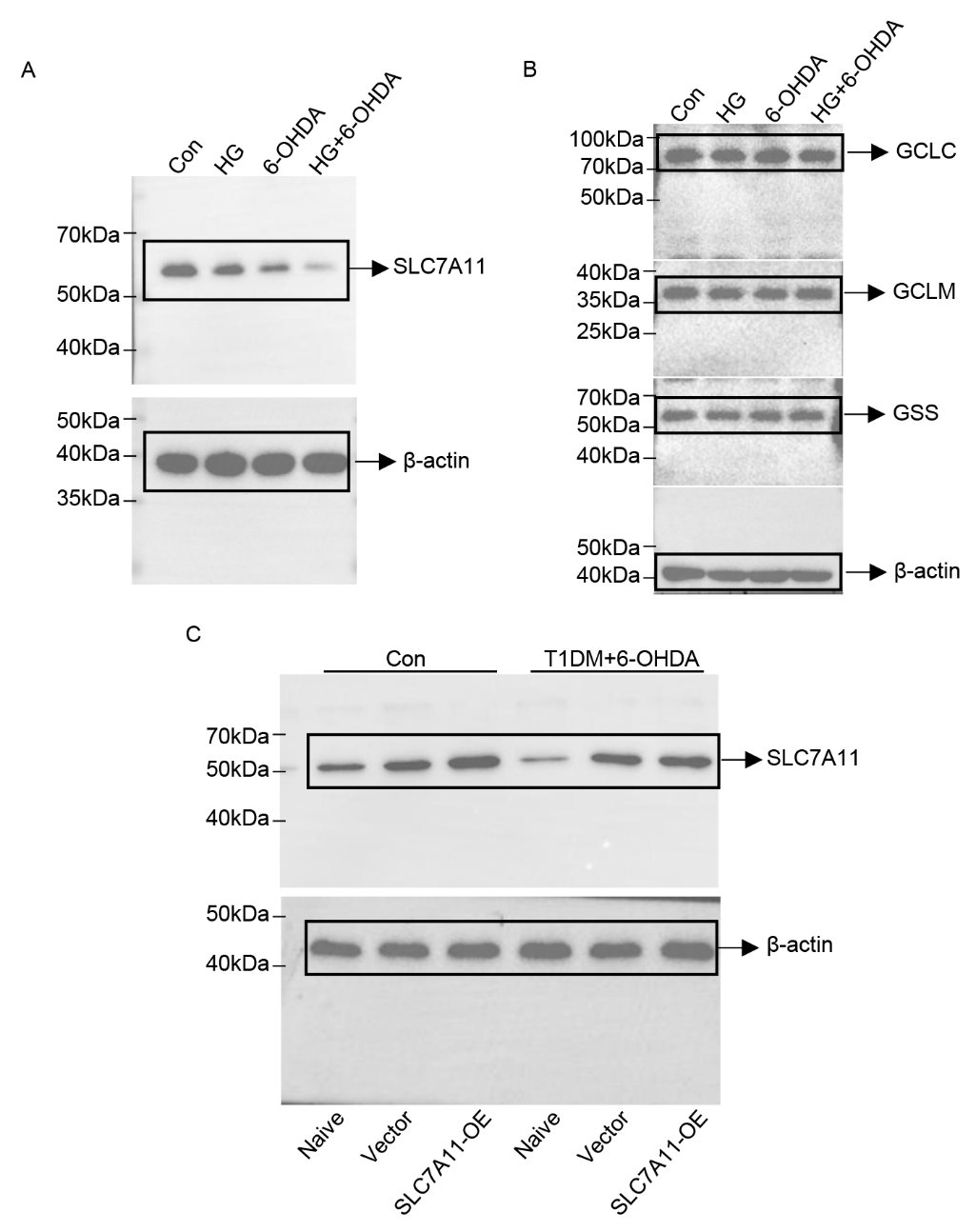


Supplementary Figure 2. Uncropped figures from western blots. Uncropped western blot images that correspond to Figure 4C (A), Figure 4E (B), Figure 7A (C).
